# Plasma metabolomics for the diagnosis and prognosis of H1N1 influenza pneumonia

**DOI:** 10.1186/s13054-017-1672-7

**Published:** 2017-04-19

**Authors:** Mohammad M. Banoei, Hans J. Vogel, Aalim M. Weljie, Anand Kumar, Sachin Yende, Derek C. Angus, Brent W. Winston, the Canadian Critical Care Translational Biology Group (CCCTBG)

**Affiliations:** 1 Department of Critical Care Medicine, University of Calgary, Calgary, AB Canada; 2 Bio-NMR Centre, Centre for Advanced Technologies, Department of Biochemistry and Molecular Biology, University of Calgary, Calgary, AB Canada; 3 Department of Pharmacology, University of Pennsylvania, Philadelphia, PA USA; 4 Section of Critical Care Medicine, Winnipeg Health Sciences Centre, St. Boniface Hospital, University of Manitoba, Winnipeg, MB Canada; 5 Clinical Research, Investigation, and Systems Modeling of Acute Illness (CRISMA) Center, Department of Critical Care Medicine, University of Pittsburgh, Pittsburgh, PA USA; 6 Department of Critical Care Medicine, University of Pittsburgh, Pittsburgh, PA USA; 7 Department of Medicine, University of Calgary, Health Research Innovation Center (HRIC), Room 4C64, 3280 Hospital Drive N.W., Calgary, AB T2N 4Z6 Canada; 8 Department of Biochemistry and Molecular Biology, University of Calgary, Health Research Innovation Center (HRIC), Room 4C64, 3280 Hospital Drive N.W., Calgary, AB T2N 4Z6 Canada

**Keywords:** H1N1 pneumonia, Metabolomics, NMR, GC-MS, Biomarkers

## Abstract

**Background:**

Metabolomics is a tool that has been used for the diagnosis and prognosis of specific diseases. The purpose of this study was to examine if metabolomics could be used as a potential diagnostic and prognostic tool for H1N1 pneumonia. Our hypothesis was that metabolomics can potentially be used early for the diagnosis and prognosis of H1N1 influenza pneumonia.

**Methods:**

^1^H nuclear magnetic resonance spectroscopy and gas chromatography-mass spectrometry were used to profile the metabolome in 42 patients with H1N1 pneumonia, 31 ventilated control subjects in the intensive care unit (ICU), and 30 culture-positive plasma samples from patients with bacterial community-acquired pneumonia drawn within the first 24 h of hospital admission for diagnosis and prognosis of disease.

**Results:**

We found that plasma-based metabolomics from samples taken within 24 h of hospital admission can be used to discriminate H1N1 pneumonia from bacterial pneumonia and nonsurvivors from survivors of H1N1 pneumonia. Moreover, metabolomics is a highly sensitive and specific tool for the 90-day prognosis of mortality in H1N1 pneumonia.

**Conclusions:**

This study demonstrates that H1N1 pneumonia can create a quite different plasma metabolic profile from bacterial culture-positive pneumonia and ventilated control subjects in the ICU on the basis of plasma samples taken within 24 h of hospital/ICU admission, early in the course of disease.

**Electronic supplementary material:**

The online version of this article (doi:10.1186/s13054-017-1672-7) contains supplementary material, which is available to authorized users.

## Background

H1N1 influenza infection is a major health burden that can be life-threatening, particularly among the elderly and patients with comorbid diseases [1, 2]. In adults, influenza remains the predominant viral cause of community-acquired pneumonia (CAP) and has a relatively high mortality [3, 4]. The case fatality rate of influenza pneumonia in adults can reach up to 30% in the intensive care unit (ICU). Approximately 5% to 9% of patients with influenza in the United States, and 11% in Canada, require hospitalization. Furthermore, 13% to 45.3% of hospitalized patients with influenza pneumonia are admitted to the ICU [5]. It is noteworthy that from 12 April 2009 to 10 April 2010, there were approximately 60.8 million cases of H1N1 (range 43.3 million–89.3 million) resulting in 274,304 hospitalizations (range 195,086–402,719) and 12,469 deaths (range 8868–18,306) in the United States [6].

Early identification of patients with H1N1 influenza pneumonia can play a critical role in disease management by improving the early administration of antiviral drugs. Delay in therapy for H1N1 influenza pneumonia has been associated with increased ICU admission and mortality [7].

Biomarkers may facilitate early diagnosis and prognosis, as well as help determine response to treatment and develop new insights into ongoing pathophysiologic processes in viral pneumonia. One promising approach for identifying biomarkers of disease is the use metabolomic profiling. The application of metabolomics in the investigation of various diseases has rapidly evolved and provides researchers with a powerful approach to gain new insights into the pathophysiologic mechanisms of disease and enhance diagnostic and prognostic tools [8].

Targeted and nontargeted metabolomic methods such as proton nuclear magnetic resonance (^1^H-NMR) spectroscopy, gas chromatography-mass spectrometry (GC-MS), and liquid chromatography-mass spectrometry allow for the identification of more than 4000 metabolites in human biofluids [9]. ^1^H-NMR and GC-MS analyses of biofluids are widely used as potential tools with highly reproducible results for the identification of metabolites [10].

Using nontargeted ^1^H-NMR and GC-MS approaches, we tested the hypothesis that metabolomic profiling can be applied to plasma samples drawn within 24 h of admission to the hospital to diagnose patients with H1N1 pneumonia vs. patients with bacterial CAP and ventilated ICU control subjects. In addition, we further hypothesized that plasma metabolomics could be used for the prognosis of mortality through separation of H1N1 nonsurvivors from survivors using samples drawn within 24 h of hospital admission.

## Methods

### Study subjects

Forty-two patients with confirmed influenza virus A (H1N1) pneumonia were included in this study from multiple Canadian centers by the University of Manitoba. Only patients ≥18 years of age were included in the study.

To diagnose H1N1, 31 noninfected ventilated control ICU patients were selected on the basis of age- and sex-matching to the patients with H1N1 (Table 1). Ventilated ICU control subjects were patients admitted to the ICU postoperatively after an elective procedure (e.g., posterior cranial fossa or spinal surgery) if there was no suspicion of infection and plasma was collected on day 1 of ICU admission while these patients were ventilated in the ICU. Moreover, 29 culture-positive samples from patients with bacterial CAP were selected from a multicenter study at the University of Pittsburgh for diagnostic comparison of bacterial pneumonia with the patient cohort with H1N1 virus (Table 1). Bacterial CAP samples were identified on the basis of clinical and radiologic criteria as described previously [11]. The bacterial sources of infection included different species, such as *Streptococcus pneumoniae*, *Staphylococcus aureus*, *Pseudomonas aeruginosa*, and *Escherichia coli*.

**Table 1:** Clinical and demographic characteristics of H1N1 patients vs. positive bacterial culture patients and ICU ventilated controls

Variable	H1N1 patients (*n* = 29)	Positive bacterial culture (*n* = 29)
Age yrs. (mean ± SD)	51.1 ± 13.2	70.4 ± 20.7
Male/Female	13/16	13/16
APACHE II/APACHE III^a^	23.5 ± 7.4	72.8 ± 24.4
ICU LOS*	20.1 ± 14.5	1.7 ± 3.2
Hospital LOS*	32 ± 23.8	10.4 ± 10.3
Smoker	13	17
Altered LOC	2	5
Renal Failure	2	1
CHF	3	6
Alcohol	3	4
Cerebrovascular Disease	3	4
Variable	H1N1 patients (*n* = 42)	ICU ventilated controls (*n* = 31)
Age yrs. (mean ± SD)	45.7 ± 14.6	50.6 ± 13.8
Male/Female	13/29	14/17
BMI (mean ± SD)*	35.2 ± 12.6	30.7 ± 7.6
APACHE II	22 ± 7.7	19.1 ± 5.7
ICU LOS*	20.6 ± 15.6	4 ± 4.9
Hospital LOS	31.5 ± 23.5	24.7 ± 33.9

*SD* standard deviation, (%) of subjects, unless otherwise indicated, *LOS* Length of Stay, *DBA* Days before admission, *APACHE II* Acute Physiology and Chronic Health Evaluation II ICU scoring system, *CHF* congestive heart disease. ^a^The APACHE II score is for H1N1 and APACHE III is for positive bacterial culture, *reflects statistically significant difference in groups *p* < 0.05

Of 42 patients with H1N1, 21 patients consisting of 7 nonsurvivors and 14 survivors were used for the mortality prognosis training set in H1N1 pneumonia. Table 2 shows the demographics of the nonsurvivors and survivors used for the training set for the mortality prognosis portion of the study.

**Table 2:** Clinical and demographic characteristics of H1N1 patients with laboratory-confirmed influenza H1N1 infection

Variables	Non-survivor H1N1 patients (*n* = 7)	Survivor H1N1 patients (*n* = 14)
Age yrs. (mean ± SD)	51.4 ± 18.3	50.2 ± 13.2
Male/Female	2/5	4/10
BMI (mean ± SD)	32.8 ± 11.7	35.2 ± 12.8
Race		
Caucasian First nation	4 2	11 3
APACHE II	23.2 ± 9.1	20.8 ± 8.3
ICU LOS	18.4 ± 7.8	18.5 ± 16.6
Symptoms DBA	5.2 ± 3.3	5.9 ± 3.2
Smoker	2 (28)	5 (35)
Alcoholism	2 (28)	2 (14)
Pregnancy	0 (0)	2 (14)

*SD* standard deviation, (%) of subjects, unless otherwise indicated, *LOS* Length of Stay, *DBA* Days before admission, *APACHE II* Acute Physiology and Chronic Health Evaluation II ICU scoring system, *CHF* congestive heart disease. ^a^The APACHE II score is for H1N1 and APACHE III is for positive bacterial culture, *reflects statistically significant difference in groups *p* < 0.05

### Study design

This case-control study was nested within three cohorts enrolled at the universities of Calgary, Manitoba, and Pittsburgh. To determine whether metabolomics can be used to diagnose H1N1 pneumonia, we compared 42 patients with H1N1 pneumonia with 31 age- and sex-matched ICU patients who required mechanical ventilation and 29 patients with H1N1 pneumonia with 29 sex-matched patients with bacterial CAP. To determine whether metabolomics could be used to predict mortality among 42 patients with H1N1, we compared 14 survivors and 7 age- and sex-matched nonsurvivors (ratio 2:1) at 90 days as a training set. A total of 42 patient samples were examined, with 21 patient samples used as a “discovery” cohort for the mortality study. The other 21 patient samples were used as a validation cohort; however, all of these were survivors. All tested patients with H1N1 infection had no initial detected bacterial coinfection.

### ^1^H-NMR spectroscopic analysis and metabolite concentration profiling

^1^H-NMR spectroscopic analysis was performed in one-dimensional mode for all samples using a 600-MHz Bruker Ultrashield Plus NMR spectrometer (Bruker BioSpin Ltd., Milton, ON, Canada). Details of the pulse sequence can be found in the data acquisition section of the supplement (see Additional file 1). Chenomx NMR Suite 7.1 software (Chenomx Inc., Edmonton, AB, Canada) was used to profile the ^1^H-NMR spectra for metabolite identification and quantification using a nontargeted profiling approach in the profiler module [12]. We used 4,4-dimethyl-4-silapentane-1-sulfonic acid as an internal standard for metabolite quantification [13].

### GC-MS

GC-MS analysis was also performed on all samples using an Agilent chromatograph 7890A (Agilent Technologies, Santa Clara, CA, USA) coupled with a Waters GCT mass spectrometer (Waters Corp., Milford, MA, USA), using a gas chromatography time-of-flight mass spectrometry technique. The mass spectrometer was programmed in the range of 50–800 mass-to-charge ratio. Using Metabolite Detector software (version 2.06; Institut für Biochemie & Biotechnologie, Technische Universität Carolo-Wilhelmina zu Braunschweig, Braunschweig, Germany), mass spectra were processed and analyzed to detect compounds. The Golm Metabolome Database [14] and National Institute of Standards and Technology 2008 library [15] were used to identify compounds. For sample preparation information, please see the online supplement (see Additional file 1).

### Multivariate data analyses

Unsupervised multivariate principal component analysis (PCA) was performed to assess the data acquired on plasma ^1^H-NMR metabolites and GC-MS features from patients with H1N1 (*n* = 42) vs. ventilated ICU control subjects (*n* = 31) and from patients with H1N1 (*n* = 29) vs. patients with CAP with positive bacterial cultures (*n* = 29). PCA was also performed as an exploratory model on all identified plasma ^1^H-NMR and GC-MS data from the training set of H1N1 samples (14 H1N1 survivors vs. 7 nonsurvivors matched by age and sex). The PCA model was used to identify data grouping and outliers and to examine the intrinsic differences between the two cohorts for each analytical technique. The number of quantified metabolites/features for PCA analysis of each study are depicted in Additional file 2: Table S14.

Orthogonal partial least-squares discriminant analysis (OPLS-DA) was then performed to build prediction models of H1N1 diagnosis and mortality. OPLS-DA was used to maximize covariance between the measured variables and the response variables (predictive classifications). The quality of the OPLS-DA model was verified using three performance indicators: cross-validation analysis of variance (CV-ANOVA), R^2^Y, and Q^2^Y (see Additional file 1 for more details). Additionally, the other parameters used to describe the predictive models were sensitivity, specificity, and AUROC. Potential confounders (e.g., age, sex, body mass index [BMI]) and comorbidities (e.g., asthma, chronic obstructive pulmonary disease [COPD]) were examined for their importance using orthogonal 2 partial least squares (O2PLS) modeling of mortality of H1N1 pneumonia.

Pathway analysis of potential biomarkers was performed using MetaboAnalyst software (http://www.metaboanalyst.ca). We also used Ingenuity Pathway Analysis (IPA) software (version 3.1; Ingenuity Systems Inc., Qiagen Bioinformatics, Redwood City, CA, USA) to discover the most important biological networks using significantly altered metabolites between cohorts. The significantly changed metabolites were obtained through multivariate data analysis; OPLS-DA was used to discriminate metabolomic profiles between two groups.

### Prediction set modeling

To obtain sensitivity, specificity, and AUROC data, we performed a prediction test to create a misclassification table for all discriminant analysis models for diagnosis and prognosis studies. We repeated the process three times to create randomly the prediction test and to average sensitivity and specificity. Moreover, for the prognosis of mortality study, the prediction sets were created from current active models (work sets) by taking seven random samples (two nonsurvivors and five survivors) out of the OPLS-DA models three times and averaging sensitivity, specificity, and AUROC. In addition, another prediction model (validation) was tested using the cohort of 21 survivors who were not used in the former prediction (test) set and 2 randomly selected nonsurvivors. Misclassification tests showed 100% sensitivity and 100% specificity for this prediction group.

### Univariate data analysis

Univariate analysis was performed as a complementary method to multivariate statistical analysis for enhancing the amount of information from the study and to serve as a less complex way to understand the cohort differences. We used MetaboAnalyst software for the univariate analysis and Student’s *t* test for evaluation of each variable individually to determine whether the means of two groups were distinct for the diagnosis and prognosis studies. To perform univariate analysis, both NMR and GC-MS datasets were normalized, followed by log transformation and autoscaling. The important features in each dataset were selected by *t* test with a threshold of 0.05.

## Results

### Diagnosis of H1N1 pneumonia based on a ventilated ICU control population and patients with culture-positive bacterial CAP

To assess the value of plasma metabolomics for the diagnosis of H1N1 pneumonia, patients with H1N1 pneumonia, ventilated ICU control subjects, and patients with CAP with positive bacterial cultures were used to explore and create prediction models based on 29 patients with H1N1 pneumonia vs. 29 sex-matched patients with CAP with positive bacterial cultures and based on 42 patients with H1N1 pneumonia vs. 31 age- and sex-matched ventilated ICU control subjects (Table 1).

### Metabolomic pattern of diagnostic cohorts

**Fig. 1:**
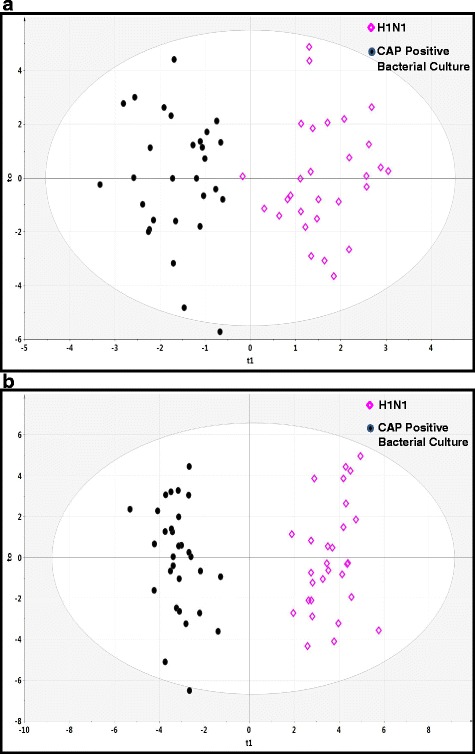
The orthogonal partial least-squares discriminant analysis (OPLS-DA) of patients with H1N1 vs. patients with CAP with positive bacterial cultures showing the best possible discrimination. **a** OPLS-DA plot for proton nuclear magnetic resonance data (*R* ^2^ = 0.824, *Q* ^2^ = 0.657, *P* < 0.0001). **b** OPLS-DA plot for gas chromatography-mass spectrometry data (*R* ^2^ = 0.937, *Q* ^2^ = 0.879, *P* < 0.0001). The *x*-axis is the prediction component that shows differences between groups, and the *y*-axis shows the orthogonal component differences within the group. *R* ^2^ represents goodness of fit, *Q* ^2^ represents a goodness of prediction, and the *P* value shows the significance level of the model (*x*-axis = predictive components, *y*-axis = orthogonal component)

**Fig. 2:**
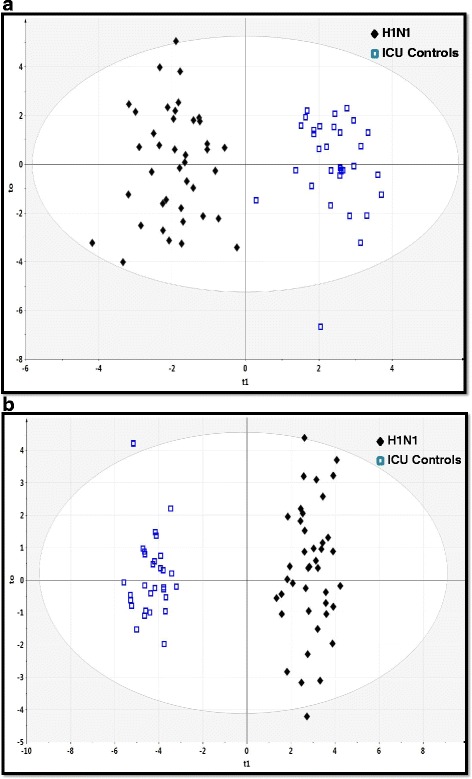
The orthogonal partial least-squares discriminant analysis (OPLS-DA) of patients with H1N1 vs. ventilated ICU control subjects shows the best possible discrimination. **a** OPLS-DA plot for proton nuclear magnetic resonance data (*R* ^2^ = 0.889, *Q* ^2^ = 0.789, and *P* < 0.0001). **b** OPLS-DA plot for gas chromatography-mass spectrometry data (*R* ^2^ = 0.963, *Q* ^2^ = 0.946, *P* < 0.0001). The *x*-axis represents the prediction component that shows differences between groups, and the *y*-axis represents the orthogonal component differences within the group. *R* ^2^ represents goodness of fit, *Q* ^2^ represents goodness of prediction, and *P* value shows the significance level of the model (*x*-axis = predictive components, *y*-axis = orthogonal component)

A PCA scatterplot of the entire ^1^H-NMR and GC-MS datasets demonstrated that the H1N1 cohort could be distinguished from patients with CAP with positive bacterial cultures (see Additional file 2: Figure S1a and b) and the ventilated ICU control subjects (see Additional file 2: Figure S2a and b). OPLS-DA models showed a clear discrimination of metabolomic profiles of patients with H1N1 from profiles of patients with CAP with positive bacterial cultures (Fig. 1a and b) and ICU control subjects (Fig. 2a and b).

Table 3 shows the prediction models using OPLS-DA to discriminate plasma H1N1 samples from ICU control subject and positive bacterial culture CAP samples on the basis of NMR and GC-MS datasets and number of metabolites that contributed in the separation between groups. *Q* ^2^ indicates an excellent separation between the plasma metabolic profile of H1N1 and ICU control subjects, whereas the prediction model for the NMR dataset is not as high as the GC-MS dataset to separate H1N1 from positive bacterial culture CAP cohort data. There are a large number of metabolites contributing to the separation of patients with H1N1 from patients with CAP with positive bacterial cultures and ventilated ICU control subjects, suggesting that H1N1 pneumonia could create a disease-specific metabolic profile quite distinct from the two other cohorts (Table 4). These data show that plasma metabolomics using ^1^H-NMR and GC-MS analytical platforms could be applied as a diagnostic tool with high predictability, sensitivity, and specificity for identification of H1N1 influenza pneumonia. The data show that H1N1 pneumonia is accompanied by metabolomic changes in the concentration of some common metabolites (amino acids and ketone bodies) and some specific metabolites compared with patients with CAP with positive bacterial cultures and ventilated ICU control subjects. We also observed that some metabolites had similar patterns of changes in H1N1 in comparison to samples of patients with bacterial CAP and ICU control subjects. H1N1 pneumonia samples showed increased concentrations of dimethylamine, β-alanine, formate, and quinic acid and a decreased  concentration of alanine vs. the two other cohorts. Overall, plasma metabolic profiles by important metabolites/features showed clear differences between diagnosis of H1N1 compared to positive bacterial culture and diagnosis of H1N1 compared to ventilated ICU control subjects (Table 4).

**Table 3:** Summary of discrimination (OPLS-DA) modeling statistics for the diagnostic and prognosis of H1N1

Analytical tool	R^2^Y	Q^2^Y	*P* value	Sensitivity	Specificity	AUROC	Metabolites/features
Discrimination (OPLS-DA) models for differentiation of H1N1 (*n* = 29) from sex-matched positive bacterial culture CAP samples (*n* = 29)							
NMR	0.825	0.589	<0.0001	87	100	0.908	50
GC-MS	0.937	0.879	<0.0001	100	100	0.942	70 (known)
Discrimination (OPLS-DA) models for differentiation of H1N1 (*n* = 42) from age- and sex-matched ICU controls (*n* = 31)							
NMR	0.889	0.789	<0.0001	100	100	0.921	55
GC-MS	0.981	0.971	<0.0001	100	100	0.959	68 (37 known)
Discrimination (OPLS-DA) models for prognosis of 90-day mortality in H1N1 (non-survivors (*n* = 7) vs. age- and sex-matched survivors (*n* = 14)							
NMR	0.831	0.597	0.004	100	100	0.865	20
GC-MS	0.909	0.829	0.0001	100	100	0.909	63 (32 known)

**Table 4:** Most significant metabolites/ features changed between two cohorts for diagnosis and prognosis studies

	Diagnosis of H1N1 pneumonia from positive bacterial culture pneumonia		Diagnosis of H1N1 pneumonia from ventilated ICU controls		Prognosis of mortality of H1N1 pneumonia (non-survivors vs. survivors)	
	Decreased in H1N1	Increased in H1N1	Decreased in H1N1	Increased in H1N1	Decreased in H1N1 nonsurvivors	Increased in H1N1 nonsurvivors
NMR	Citrate Fumarate 3-Methyl,2-Isovalerate Alanine Tyrosine Methionine Histidine 4-Hydroxybutyrate	Acetoacetate Beta-alanine Formate Dimethylamine Carnitine Glycine	Isopropanol Citrate Taurine Glycine 2-Oxoglutarate Glutamine Alanine Serine	Dimethylamine Beta-alanine Aspartate Phenylalanine Formate 3-Hydroxyisovalerate Fumarate O-Phosphocholine Adipate Choline 2-Hydroxyisovalerate Proline Ornithine	2-Oxoglutarate Dimethylamine Isopropanol Carnitine 2-Hydroxisovalerate Lactate Phenylalanine Acetate Tyrosine	2-Aminobutyrate Acetoacetate 2-Hydroxybutyrate Arginine 3-Hydroxybutyrate
GC-MS^a^	Uric acid Tyrosine Citric acid Asparagine Myoinositol Lysine Arabinonic acid Threonine Aspartic acid Threonic acid	Gulonic acid Pentadecane 2-amino Butanoic acid Alkane Quinic acid Benzoic acid	Galactose Lactic acid Glucose Pyroglutamic acid Galactopyranoside Glyceric acid Fructose Beta alanine Glycerol Glycine 3-hydroxyl Butanoic acid Phenylalanine	Heptadecanoic acid Phosphoric acid Hexadecanoic acid Octadecenoic acid Octadecane Quinic acid	Threonic acid Dodecane Decanoic acid 2-Amino, Butanoic acid Valine Glycerol	Methionine Pentadecane 4, Amino, Benzoic acid Hydroxylamine

^a^The unknown’s features have not been listed

Coefficient plots revealed the relative correlation of metabolites and features that showed decreased and increased concentrations in nonsurvivors vs. survivors and in patients with H1N1 vs. ICU control subjects on the basis of NMR and GC-MS datasets. Table 5 shows the most important biological pathways for diagnosis and prognosis based on multivariate data analysis using coefficient plot and S-plot analysis. S-Plot analysis was used to identify putative biomarkers on the basis of related OPLS-DA models to choose metabolites/features with high magnitude and high reliability (see Additional file 2: Figures S6, S8 and S10).

**Table 5:** Top biological pathways based on identified metabolites involved for diagnosis of and prognosis of H1N1

MetaboAnalyst Pathway analysis	NMR	GC-MS^a^
Diagnosis of H1N1 Pneumonia from Positive Bacterial Culture Pneumonia	- Synthesis and degradation of ketone bodies - Beta-alanine metabolism - Glycine, serine and threonine metabolism - Methane metabolism - Glyoxylate and dicarboxylate metabolism - Histidine metabolism	- Lysine degradation - Inositol phosphate metabolism
Diagnosis of H1N1 Pneumonia from Ventilated ICU Controls	- Taurine and hypotaurine metabolism - Glycine, serine and threonine - Beta-Alanine metabolism - Citrate cycle (TCA cycle) - Glyoxylate and dicarboxylate metabolism - Phenylalanine metabolism	- Galactose metabolism - Glycine, serine and threonine metabolism - Pyruvate metabolism - Phenylalanine metabolism
Prognosis of mortality of H1N1 pneumonia (separation nonsurvivors from survivors)	- Synthesis and degradation of ketone bodies - Arginine and Ornithine metabolism - Arginine and Proline metabolism - Phenylalanine metabolism - Citrate cycle (TCA cycle)	- Beta-Alanine metabolism - Galactose metabolism - Alanine, Aspartate and glutamate metabolism - Glycerolipid metabolism - Pyruvate metabolism and glycolysis or glycogenesis

^a^Top biological pathways obtained using the knowns features

### Metabolic profile by univariate analysis (diagnosis study)

**Fig. 3:**
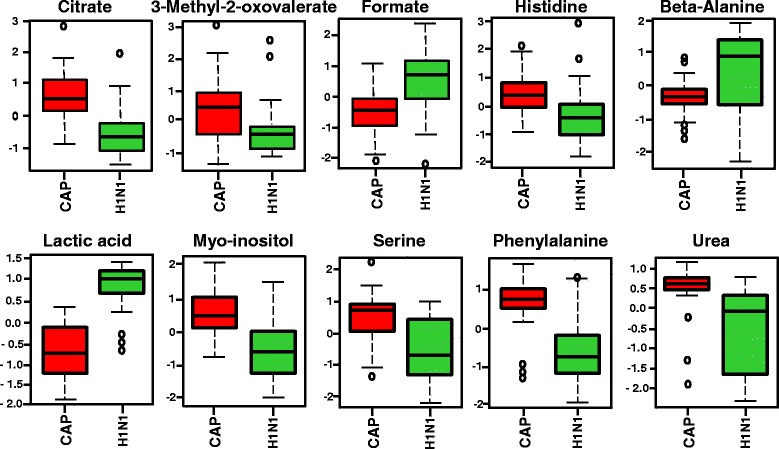
Univariate analysis showing important metabolites/features between samples of patients with H1N1 pneumonia and bacterial pneumonia samples. The top ten metabolites detected by proton nuclear magnetic resonance (*first line*) and gas chromatography-mass spectrometry (*second line*) that have significantly changed in plasma between samples of patients with H1N1 pneumonia and culture-positive bacterial pneumonia samples (units in normalized and scaled concentrations). The *x*-axis shows the specific metabolite, and the *y*-axis is the relative concentration when samples of patients with community-acquired pneumonia are compared with the samples of patients with H1N1. The box-and-whisker plots show the mean and SD of the metabolite

**Fig. 4:**
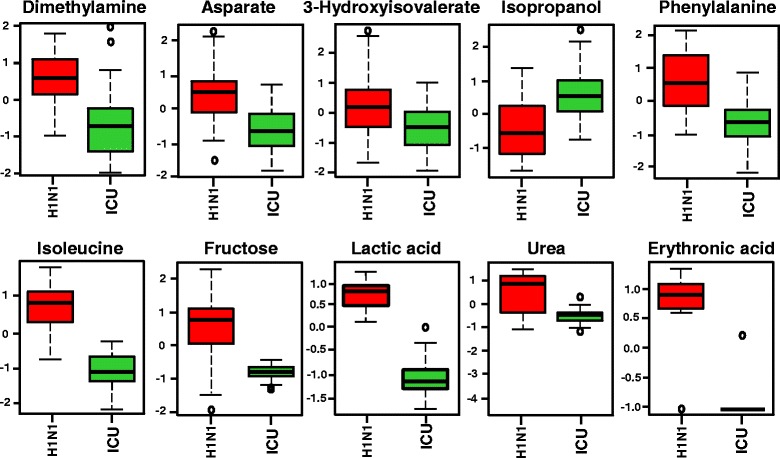
Univariate analysis showing important features of metabolites between H1N1 pneumonia samples and ventilated ICU control samples. The top ten metabolites/features detected by proton nuclear magnetic resonance (*first line*) and gas chromatography-mass spectrometry (*second line*) that have significantly changed in the plasma between H1N1 pneumonia samples and ventilated ICU control samples (units in normalized and scaled concentrations). The *x*-axis shows the specific metabolite, and the *y*-axis is the relative concentration when samples of patients with H1N1 are compared with the ventilated ICU control patient samples. The box-and-whisker plots show the mean and SD of the metabolite

Univariate analysis was performed to show the mean, SD, and *P* values of metabolites/features obtained by NMR and GC-MS. Additional file 2: Tables S1 and S2 show the significantly different metabolites (*P* < 0.05) between H1N1 pneumonia and positive bacterial culture pneumonia samples for NMR (*n* = 13) and GC-MS (*n* = 98). Additional file 2: Tables S3 and S4 also show the significantly different metabolites (*P* < 0.05) between patients with H1N1 pneumonia and ventilated ICU control subjects for NMR (*n* = 27) and GC-MS (*n* = 57). Multivariate data analysis revealed more metabolites that were significantly changed between the H1N1 pneumonia cohorts and the two other cohorts on the basis of NMR data, but the GC-MS findings did not show a large difference in the number of metabolites between multivariate and univariate data analyses. Although the multivariate and univariate methods revealed high numbers of overlapping metabolites/features, we found a different pattern for more significant metabolites/features between multivariate and univariate methods in the diagnosis of H1N1 (Figs. 3 and 4).

### Prognosis of mortality of H1N1

For the prognosis of 90-day mortality, prediction models were built on the basis of the training set. There were no statistically significant differences (*P <* 0.05) on tested demographic variables between the survivor and the nonsurvivor cohorts, except for the presence of fever, which was higher in the survivor cohort (see Additional file 2: Table S15).

### Metabolomic pattern of the prognostic cohorts

A PCA score plot of the entire ^1^H-NMR dataset based on the first and second principal components demonstrated that the 90-day nonsurvivor group could be distinguished from the survivor group; that is, there was data clustering) (see Additional file 2: Figure S3a). The PCA score plot of the features detected by GC-MS revealed similarly separated clusters for H1N1 nonsurvivors and survivors (see Additional file 2: Figure S3b).

**Fig. 5:**
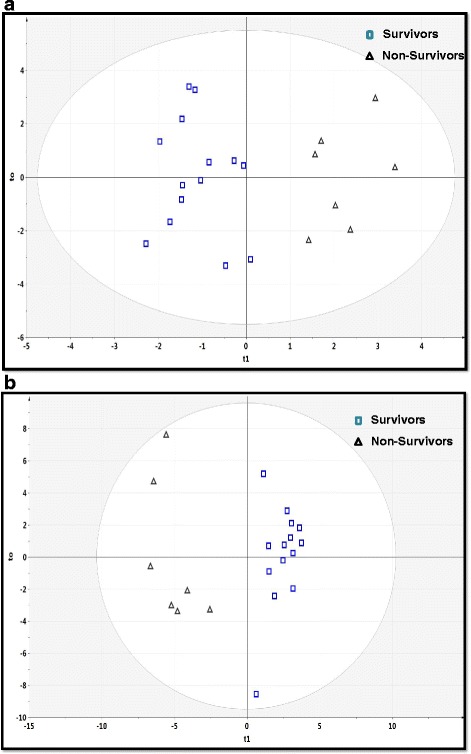
The supervised orthogonal partial least-squares discriminant analysis (OPLS-DA) shows the best possible discrimination between nonsurvivors and survivors of H1N1 infection. **a** OPLS-DA plot for proton nuclear magnetic resonance data (*R* ^2^ = 0.831, *Q* ^2^ = 0.597, *P* = 0.004). **b** OPLS-DA plot for gas chromatography-mass spectrometry data (*R* ^2^ = 0.909, *Q* ^2^ = 0.829, *P* = 0.0001). The *x*-axis represents the prediction component that shows differences between groups, and the *y*-axis represents the orthogonal component differences within the group. *R* ^2^ represents goodness of fit, Q^2^ represents goodness of prediction, and *P* value shows the significance level of the model (*x*-axis = predictive components, *y*-axis = orthogonal component)

Once data clustering was revealed, we analyzed the metabolomic profiling data using supervised OPLS-DA for modeling. Of the NMR data (Fig. 5a), 27 different metabolites were used as potential variables (metabolites) to separate nonsurvivors from survivors with a R^2^Y = 0.831 and a Q^2^Y = 0.597, indicating very good separation between the two cohorts at the plasma metabolomic level for H1N1 mortality (Table 3). We used a statistical approach based on variable importance in the projection ≥1 to determine the number of analytes for the best predictive model using SIMCA-P Version 13.0, Umetrics AB, Umea, Sweden) [16].

As shown in Fig. 5b, the GC-MS OPLS-DA model showed samples from the survivor and nonsurvivor cohorts were well separated on the basis of 63 features (32 known and 31 unknown). The model characteristics were numerically significant, with a R^2^Y = 0.909 and a Q^2^Y = 0.829, indicating an excellent model. To assess the reliability of the OPLS-DA models for the NMR and GC-MS data, CV-ANOVA was performed. The *P* values for both models were 0.004 and 0.0001 for the NMR and GC-MS models, respectively.

Using O2PLS discriminant analysis [17], we tested the role of confounding factors in separation of nonsurvivors from survivors, including patient characteristics and comorbidities, but these did not affect the observed discrimination to prognosticate the mortality for both NMR and GC-MS analyses. This suggests that the differentiation between the survivor and nonsurvivor groups is based on disease metabolite changes rather than on the roles of age, sex, and BMI in this study (see Additional file 2: Figure S4a–c). Given these results, it can be concluded that ^1^H-NMR and GC-MS are appropriate analytical tools to apply a metabolomic approach for prognosis of 90-day mortality of patients with H1N1 influenza pneumonia on the basis of samples taken within 24 h of hospitalization.

### Metabolic profile by univariate analysis (prognosis study)

**Fig. 6:**
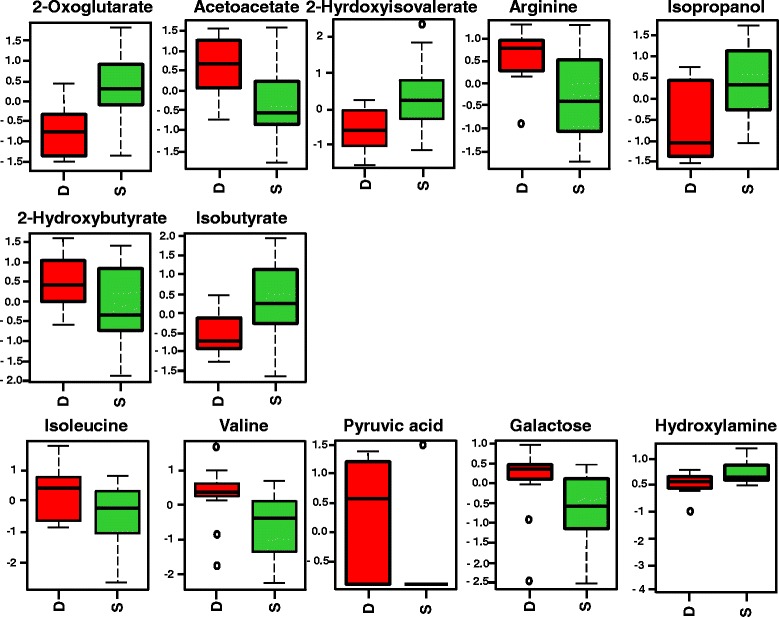
Univariate analysis showing all metabolites/features between H1N1 pneumonia nonsurvivors and H1N1 pneumonia survivors. All significant metabolites/features detected by proton nuclear magnetic resonance (*first* and *second lines*) and gas chromatography-mass spectrometry (*third line*) that have significantly changed in the plasma between H1N1 pneumonia nonsurvivors (D) and H1N1 pneumonia survivors (S) (units in normalized and scaled concentrations). The *x*-axis shows the specific metabolite, and the *y*-axis is the relative concentration when samples from surviving patients with H1N1 are compared with the samples of nonsurviving patients with H1N1. The box-and-whisker plots show the mean and SD of the metabolite

Univariate analysis showed that normalized concentrations of 7 metabolites detected by ^1^H-NMR and the relative intensities of 19 features detected by GC-MS varied significantly among nonsurvivors and survivors. The summary of all significant metabolites/features (*P* < 0.1) from NMR and GC-MS are listed in Additional file 2: Tables S6 and S7 with their *P* values and mean (±SD) values of variables in each group. A comparison of multivariate and univariate approaches shows the differences in type and number of metabolites for both NMR and GC-MS datasets to separate nonsurvivors from survivors (see Additional file 2: Tables S5 and S6). It was interesting to note that using the multivariate statistical technique, we observed more potential metabolites/features than in univariate analysis to distinguish H1N1 nonsurvivor from survivor cohorts, with these two analytical methods showing a very different metabolic profile pattern. Univariate methods are used to simplify the interpretation of discriminating metabolites individually. Interestingly, this study showed that the univariate analysis could reject potential metabolites with only small changes that failed to have significant differences by *t* test, whereas they could be more important when they were analyzed simultaneously with the other metabolites; that is, the metabolites may not act independently on the outcome. Figure 6 shows all metabolites/features that are significantly different between nonsurvivors and survivors detected by NMR and GC-MS platforms using univariate analysis.

### Metabolic pathway and function analysis

The important potential targets of metabolic pathway analysis obtained using MetaboAnalyst for NMR and GC-MS data showed an impact value ≥0.10. More detailed analysis of the most relevant pathways is listed in Addtitional file 2: Tables S7-S12 and Figures S11–S13. Table 5 shows the identified biological pathways involved, based on the most important metabolites found by NMR and GC-MS analyses, in the diagnosis of H1N1 pneumonia based on culture-positive bacterial pneumonia and ventilated ICU control subjects and prognosis of mortality in H1N1 pneumonia. The pathways have been ordered by their impact values from high to low in Table 5. Table 5 shows more differentiated biological pathways involved in the diagnosis of H1N1 from ICU control subjects compared with patients with culture-positive bacterial CAP. This evidence suggests that the difference in metabolomic profile between patients with H1N1 pneumonia and ICU control subjects is greater than that of patients with H1N1 pneumonia and culture-positive bacterial pneumonia, based on the OPLS-DA models (Table 4) as well as on the biological pathways. It is interesting that a variety of biological pathways were found in separation of H1N1 nonsurvivors from survivors on the basis of metabolite changes. More potential networks of biological pathways were generated through the use of IPA software in the diagnosis and prognosis of mortality studies for both NMR and GC-MS datasets (Table 6).

**Table 6:** Top network of biological pathways for diagnosis and prognosis studies based on the NMR and GC-MS datasets

	Diagnosis of H1N1 pneumonia from positive bacterial culture pneumonia	Diagnosis of H1N1 pneumonia from ventilated ICU controls	Prognosis of mortality of H1N1 pneumonia (nonsurvivors vs. survivors)
NMR	1. Amino Acid Metabolism, Molecular Transport, Small Molecule Biochemistry 2. Drug Metabolism, Molecular Transport, Small Molecule Biochemistry	1. Cell Cycle, Hepatic System Development and Function, Cell-To-Cell Signaling and Interaction 2. Molecular Transport, Nucleic Acid Metabolism, Small Molecule Biochemistry 3. Drug Metabolism, Molecular Transport, Small Molecule Biochemistry	1. Increased levels of albumin. 2. Cell to cell signaling and interaction. 3. Cellular growth and proliferation mechanisms.
GC-MS^a^	1. Amino Acid Metabolism, Molecular Transport, Small Molecule Biochemistry	1. Cellular Compromise, Lipid Metabolism, Small Molecule Biochemistry 2. Lipid Metabolism, Molecular Transport, Small Molecule Biochemistry 3. Lipid Metabolism, Small Molecule Biochemistry, Cellular Assembly and Organization 4. Molecular Transport, Nucleic Acid Metabolism, Small Molecule Biochemistry.	1. Lipid metabolism. 2. Amino acid metabolism

^a^The generated networks obtained using known features

## Discussion

A number of studies show the feasibility of using metabolomics for the diagnosis and prognosis of noninfectious and infectious pulmonary diseases in humans, including asthma [18], COPD [19], tuberculosis [20], hepatitis [21], sepsis [22–24], and pneumonia [25, 26]. We have profiled the plasma metabolite response to pneumonia caused by infection with the H1N1 influenza virus, infection with bacteria, and noninfected, ventilated ICU control subjects, and we found that the metabolomic profiles strongly predict a difference between patients with H1N1 pneumonia vs. culture-positive patients with bacterial CAP and between patients with H1N1 pneumonia vs. ventilated ICU control subjects. Moreover, for H1N1 pneumonia prognosis, plasma metabolic profiles were highly specific and predictive to separate the two cohorts of H1N1 pneumonia 90-day nonsurvivors and survivors, using plasma samples taken on the first day of admission to the hospital. This study shows that plasma metabolomics can be a diagnostically and prognostically useful tool to diagnose H1N1 pneumonia and predict mortality among a ventilated ICU population early in the course of the disease.

It is important to note that although the H1N1 pneumonia and culture-positive bacterial CAP cohorts were collected using two different anticoagulants—ethylenediaminetetraacetic acid, and sodium heparin—for metabolomic profiling, there is strong evidence showing comparability [27, 28]; however, this remains a potential problem for this analysis. Pathway analysis showed that some specific biological pathways were significantly enriched in the diagnosis of H1N1 compared with culture-positive bacterial CAP samples, when comparing the diagnosis of H1N1 from ventilated ICU control subjects, and when examining the prognosis of mortality. Lysine degradation could be related to the role of lysine in various mechanisms of fatty acid metabolism. Unpublished data by the authors suggest a role of lipid-derived metabolites in the prognosis of mortality in patients with bacterial CAP. Lysine is known as an essential amino acid that is not synthesized in humans, whereas lysine is the product of meso-2,6-diaminopimelate/lysine biosynthesis pathways for protein synthesis in gram-negative and gram-positive bacteria [29], likely made available from bacteria in the gastrointestinal tract.

Bacterial infections have been shown to be associated with the elevation of a number of metabolites. For example, systemic bacterial infection is accompanied by elevation of histidine uptake in the human jejunum [30]. Moreover, histidine biosynthesis appears to occur in certain bacterial infections when compared with viral infection; in particular, pneumonia caused by chlamydia and *Acinetobacter* infections increase histidine biosynthesis [31]. Of note, histamine is a product of L-histidine metabolism, and this is a major metabolite present in inflammation and potentially in bacterial pneumonia [32]. Another lung pathogen, *P. aeruginosa*, also causes elevation of histidine catabolism [33]. In addition, elevated inositol phosphate metabolism has been observed with *Pneumocystis* pneumonia [34] and *S. aureus* infection in type II alveolar epithelial cells [35]. Methane metabolism pathways have been shown to be elevated in bacterial meningitis caused by pathogenesis of different species, such as *S. pneumoniae*, *N. meningitis*, *Haemophilus influenzae*, and *S. aureus* [36].

H1N1 infection does have effects on metabolism. In terms of differentiation of H1N1 from ventilated control subjects, taurine and hypotaurine metabolism showed high pathway impact in pathway analysis. Taurine is an important compound in bile acid conjugation in the liver, suggesting some involvement of the liver during H1N1 infection. Moreover, taurine is an important intracellular free amino acid that is known as an antioxidant and a neuromodulator and is also involved in regulation of osmolarity in the neural retina and brain. Patients with H1N1 showed lower concentration of taurine than ventilated ICU control subjects [37–39].

Glycine, serine, and threonine metabolism is a common pathway to differentiate patients with H1N1 from patients with bacterial CAP and ventilated ICU control subjects (Tables 3 and 4). For example, protein kinase R is an active protein in the H5N1 infection against the antiviral effects of a serine-threonine protein [40]. The decreased concentration of glycine, serine, and threonine in patients with H1N1 compared with ventilated ICU control subjects as well as culture-positive patients with bacterial CAP might implicate consumption of these amino acids through viral metabolism.

In the mortality evaluation, ^1^H-NMR and GC-MS pathway analyses revealed involvement of pathways associated with metabolism of glutamate, aspartate, and related compounds. When we examined these pathways in the context of acute H1N1 pneumonia deaths, they pointed to important clues in energy metabolism, glucose availability, and protein synthesis. Not surprisingly, one could hypothesize that interfering with any of these pathways would result in a worse prognosis, as appears to be the case. Pathway analysis points to the importance of energy metabolism with the involvement of glutamate, pyruvate, and alanine. On the basis of the findings of this study, it would appear that disruption of amino acid metabolism and gluconeogenesis pathways may be key factors in regulating the difference between nonsurvivor and survivor responses to H1N1 infection. Pathogenically, despite the primary involvement of the respiratory system, the liver and kidneys are also targets for viral infection, which can have significant effects on metabolism [41]; this is highlighted in the online supplement.

Chong and Street [42] observed that clinical presentations in elderly patients did not appear to be useful for prognosis of mortality, and our data support this finding. Metabolomic profiling revealed that the pathophysiologic pathways initiated or affected by H1N1 infection have a greater influence on the metabolic responses leading to mortality than the other observed factors, such as clinical demographics and serious comorbidities.

Overall, the metabolic response to H1N1 infection creates a very distinct metabolic signature compared with that related to bacterial infection and ventilated ICU control subjects, which may be exploited for diagnostic purposes and, potentially, to follow response to therapy (Table 3). As expected, a large number and variety of top networks of biological pathways are different between patients with H1N1 and ventilated ICU control subjects (Table 6).

Few studies have evaluated the diagnostic ability of metabolomics biomarkers in CAP [43]. Slupsky et al. [26] performed an NMR-based study of urine in CAP. They found that the urinary metabolic profile for pneumococcal pneumonia significantly differs from the profiles of viral and other bacterial causes of pneumonia. The same group used urine metabolic profiles for the successful diagnosis of two important causes of CAP (*S. pneumoniae* and *S. aureus*) in human and animal model studies [25, 44]. Furthermore, Laiakis et al. [32] showed that the application of serum and plasma metabolomic analysis can successfully distinguish patients with severe pneumonia from community control subjects. Thus, metabolomics is being used successfully on a research basis for CAP diagnosis in humans, although it has not been used for H1N1 pneumonia diagnosis and prognosis. The diagnosis of H1N1 pneumonia requires the presence of specific symptoms, a chest x-ray consistent with an atypical pneumonia pattern, and a diagnostic polymerase chain reaction test. This paper provides evidence for the potential use of plasma metabolomics as a further diagnostic test for H1N1 pneumonia if future validation studies confirm our findings.

While the Acute Physiology and Chronic Health Evaluation II (APACHE II) has been used to compare mortality between health care systems in the ICU, it is not a good predictor of mortality in non-ICU patients [45]. APACHE II cannot accurately predict mortality in H1N1 cohorts (see Tables 1 and 2 and Additional file 2: S15). However, we show that ^1^H-NMR and GC-MS analysis can provide a highly predictive statistical model to predict nonsurvivors from survivors of H1N1 pneumonia and that these analytical tools have high sensitivity and specificity. Furthermore, the usefulness of metabolomics for mortality prediction in H1N1 is shown using ROC curves as well as by linear regression (*R* ^2^ score) when compared with APACHE II scores of the patients (see Additonal file 2: Table S13).

There is no single, best choice of metabolomic analytical techniques, because each of these methods carries its own advantages and disadvantages [46]. Although GC-MS is a more sensitive method with high separation efficiency, high spectral resolution, and high resolution to detect compounds, NMR is more quantifiable and reproducible [46].

Examining a single time point in the pathologic process is a potential limitation of this study, like in most metabolomics studies performed to date. This can be overcome only if sequential time points are examined. For this study, only single-time-point material was available for metabolomic evaluation. Despite this limitation, the data presented are compelling.

Other potential limitations of this study include the relative small sample size and the fact that there was no prospective validation. W used as many samples as were available in Canada for this study from that time period.  We do wish more samples were available but they were not. The samples size, though small, is still of sufficient size to yield significant and compelling results. A repeat study with more patients in the future would be of value to validate our finding. Validation was done here in a case-control format and not using a separate prospective sample collection. This type of validation is acceptable but not as powerful as a separate independent validation study. These limitations are very difficult to overcome given the limited samples available for analysis in this time period.  Certainly an independent validation study would add tremendous validity to these initial findings. Like all metabolomics studies, there are technologic limitations with the chosen techniques as the NMR study component lacks sensitivity but it is quantitative and specific: the GC-MS study component, though is more sensitive, lacks in quantitative ability and in specificity because of the limitations of the available GC-MS libraries. Despite these limitations, because the two techniques show similar findings, this adds to the validity of the study and helps overcome some of the limitations.

## Conclusions

This study demonstrates that plasma metabolomics reflects a specific profile in patients with H1N1, an approach that could importantly be applied to the diagnosis and prognosis of mortality in patients with H1N1 pneumonia. We conclude that nontargeted metabolomics using ^1^H-NMR and GC-MS is highly predictive with sufficient sensitivity and specificity to prognosticate mortality by discrimination of nonsurvivors from survivors of H1N1 influenza pneumonia. Also, analysis of the metabolome can accurately be applied to identify H1N1 pneumonia cases from those with culture-positive bacterial CAP and ventilated ICU control subjects on the first day of admission to the hospital/ICU. We speculate that metabolomic studies can be used as prediction tools for timely administration of antiviral therapy and other supportive treatments that could result in better outcomes.

## Additional files

Additional file 1: Supplementary material. (DOCX 64.6 kb)

Additional file 2: Supplementary material, figures, and tables. (DOCX 1.79 mb)

## Abbreviations

APACHE II: Acute Physiology and Chronic Health Evaluation II
BMI: Body mass index
CAP: Community-acquired pneumonia
CCCTBG: Canadian Critical Care Translational Biology Group
COPD: Chronic obstructive pulmonary disease
CV-ANOVA: Cross-validation analysis of variance
GC-MS: Gas chromatography-mass spectrometry
^1^H-NMR: Proton nuclear magnetic resonance
ICU: Intensive care unit
IPA: Ingenuity Pathway Analysis
O2PLS: Orthogonal 2 partial least squares
OPLS-DA: Orthogonal partial least-squares discriminant analysis
PCA: Principal component analysis
