# Supplementary material for: Plasma metabolomics for the diagnosis and prognosis of H1N1 influenza pneumonia
Source: Crit Care. 2017 Apr 19;21:97. doi: 10.1186/s13054-017-1672-7 (PMC5397800; doi:10.1186/s13054-017-1672-7)
Supplement: Supplementary file 1 — Supplementary material. (DOCX 64.6 kb) [file 13054_2017_1672_MOESM1_ESM.docx]

**Online Supplements**

**Materials and Methods**

**Subjects**

This study was approved by the Health Research Ethic Board of the University of Manitoba, Manitoba, Canada and the Conjoint Health Research Ethics Board at the University of Calgary. The plasma samples were collected in accordance with the guideline of central laboratory manual by the University of Manitoba, Winnipeg, Canada. Samples of 5 ml of arterial or venous blood were collected using sodium fluoride EDTA vacutainer tubes, and then were centrifuged at 1150 x g for 15 minutes at 4 °C to separate blood cells from plasma. Carefully, plasma was removed from the sample, collected in 5 ml tubes, and frozen at -80ºC within 30-60 min of collection. These samples were then thawed, aliquotted and refrozen at -80ºC to be used for study.

For the H1N1 cohorts the diagnostic criteria were that of the ROSII study and reflected the racial, gender and ethnic population in Manitoba. For the H1N1 study the inclusion criteria were: 1. Patients ≥ 18 years. 2. Confirmed influenza. We used samples from only PCR confirmed cases. 3. Requirement for ICU admission due to respiratory distress or critical illness defined as one of: a) Inspired oxygen need of >50% for at least 4 hours; b) need for mechanical ventilation; or c) use/or need for inotrope or vasopressor support. 4. Negative β-HCG test or negative bedside urine test (pending a confirmatory β-HCG test for pregnancy) in women of childbearing age (12-60 years) who were in the drug study cohort (ROSII). Not all patients were in the drug study cohort allowing 2 pregnant females to be excluded from the drug study but entered into the metabolomics portion of the study with all exclusion criteria being met other than pregnancy.

Time window for inclusion: participants needed to be enrolled into the study within 36 hours following ICU admission for confirmed or suspected influenza or, if influenza is not confirmed or suspected at ICU admission, within 36 hours of order for influenza testing. The exclusion criteria were:

1. Inability to obtain consent. 2. Patients receiving more than two doses of 150 mg or higher oseltamivir in 36 hours before study entry (ROSII study). 3. Patients having received more than 3 doses of 75 mg oseltamivir within 36 hours before study entry (ROSII study). 4. Age less than 18 years, and weight less than 45 kg. 5. Unlikely to absorb enteral study drug (e.g. patients with partial or complete mechanical bowel obstruction, intestinal ischemia, infarction, and short bowel syndrome). 6. Known allergy or hypersensitivity to oseltamivir. 7. Pregnancy or breast feeding (see exceptions above). 8. Previous enrollment in current study. 9. Concurrent involvement in an RCT examining an antiviral agent including other neuraminidase inhibitors, interferon-α and/or ribavirin. 10. Chronic renal failure requiring chronic hemodialysis. 11. Severe chronic liver disease (Child-Pugh Score 11-15). 12. Anticipated death within 24 hours as judged by attending physician or local PI. 13. Patient having “do not intubate” order (a “no CPR” or “no defibrillate” or “no chest compressions” order alone was allowed). The metabolomics study was a substudy of the ROSII study.

After obtaining informed consent, 42 patients were included in this study with confirmed H1N1 pneumonia caused by influenza virus A (H1N1). Of these, 21 patients were used for the training set including seven patients who died due to H1N1 infection and fourteen age and sex-matched individuals who survived H1N1infection.

Positive bacterial culture patient samples were selected from a big cohort of CAP patient samples from the genetic and inflammatory marker study (GenIMS, Pittsburgh, PA). The CAP samples were drawn based on the higher plasma concentration of procalcitonin (PCT > 0.25ng/ml, strongly suggestive of bacterial infection) or a positive culture during the first 24 hrs of admission to hospital.

**Case identification**

Patients hospitalized with laboratory confirmed Influenza Virus A (H1N1) infection between July and September 2009 were prospectively identified in Winnipeg, Manitoba, Canada and confirmed by the Winnipeg Regional Health Authority health hospital laboratory. Viral pneumonia was defined as an acute illness with radiographic pulmonary infiltration confirmed by one clinician and one radiologist and at least two of the following: fever, cough, dyspnea, sputum production, pleuritic chest pain, or shaking chills. The selected patient characteristics in this study are summarized in Table 1. The primary outcome measure was mortality at ≤ 90 days after the onset of illness as defined by the eligibility criteria.

Positive bacterial culture samples were obtained by the University of Pittsburgh as part of a study on genetic and inflammatory markers of sepsis (GenIMS). They collected with more than 2000 samples under a multicenter study from 42 centers in USA[[1](#_ENREF_1)].

**Study design**

This observational-retrospective, nested cohort study was conducted at the University of Calgary. Plasma samples for open-profiling metabolomics were obtained from age- and sex-matched non-survivors versus survivors in a 1:2 ratio and age- and sex-matched ICU controls in a 1:1.3 ration. All tested H1N1 infected patients had no initial bacterial co-infection detected. Only patient’s ≥ 18 years of age were included in the study. A total of 42 patient samples were examined with 21 patient samples used as a ‘discovery’ cohort for the mortality study. The other 21 patient samples were used as a prediction (validation) cohort, however, all of these were survivors. Therefore, 2 non-survivor patients were randomly chosen to be used in the prediction (validation) cohort. All samples (H1N1 and ICU controls) were drawn within the first 24 hours of admission in hospital. For the metabolomics biomarker diagnostic part of the study, all 42 H1N1 patients’ samples and 31 age and sex-matched ventilated ICU control patient samples were used. Also, 29 H1N1 patient’s samples and 29 sex-matched positive bacterial culture CAP samples were analyzed to diagnose the H1N1 from bacterial causes of CAP using metabolomics profiles. The bacterial causes included different species such as *Streptococcus pneumoniae*, *Staphylococcus aureus*, *Pseudomonas aeruginosa* and *Escherichia coli*. Bacterial CAP patient plasma samples came from a cohort of CAP patients collected in Pittsburgh for the GenIMS study (Genetics and Inflammatory Markers of Sepsis Study[[1](#_ENREF_1)]). All patients in this cohort had positive culture proven bacterial CAP.

**^1^H NMR Spectroscopy**

**Sample Preparation**

Each of the plasma samples (200 µl) was defrosted from -80ºC to 4ºC and filtered via centrifuge at 12,000 x g for 1 hour at 4ºC using 3 kDa NanoSep microcentrifuge filters which were initially prewashed five times with ddH_2_O to reduce preservative contamination. The filtrates were rinsed using an additional 100 µl of D_2_O. The filtrates were then collected into clean 1.5 ml vials. The samples were adjusted to 400 µl with 80 µl of phosphate buffer (0.5 M NaH_2_PO_4_ buffer solution at pH 7.0) containing 2.5 mM 2,2-dimethylsilapentane-5-sulfonate (DSS, final concentration 0.5 mM) as an internal reference compound, 10 µl sodium azide (1 M NaN_3_) to prevent bacterial growth, and D_2_O. The pH of the samples was adjusted to 7.0 ± 0.04 at room temperature. In addition, two dimensional NMR spectra, including total correlation spectroscopy (TOCSY) and ^1^H-^13^C heteronuclear single quantum coherence spectroscopy (^1^H-^13^C HSQC), were obtained for randomly chosen samples in order to verify chemical shift assignments**.**

**Data Acquisition**

^1^H-NMR data for all the samples were generated in a blinded and randomly selected manner using an automated sample changer on a 600 MHz Bruker Ultrashield Plus NMR spectrometer (Bruker BioSPin Ltd., Canada). The one dimensional spectra were acquired using the Bruker 1D proton spectroscopy pre-saturation pulse sequence (noesypr 1d) using an optimal water suppression program and a mixing time of 100 ms [[2](#_ENREF_2), [3](#_ENREF_3)]. Initial samples from each batch were sized to ensure the half-height line width of approximately 0.7- 0.8 Hz for the DSS peak calibrated to 0.0 ppm. The NMR spectra were obtained with 1024 scans, zero filled and Fourier transformed to 128k points. Obtained NMR spectra were corrected, including line broadening, phasing, baseline correction and referencing to the DSS peak at 0.0 ppm using the Topspin software program (Bruker BioSpin Ltd., Canada). In addition, two dimensional NMR spectra, including total correlation spectroscopy (TOCSY) and ^1^H-^13^C heteronuclear single quantum coherence spectroscopy (^1^H-^13^C HSQC), were obtained for randomly chosen samples (1 in 10, total 2 samples) in order to verify chemical shift assignment.

**Metabolite Concentration Profiling**

^1^H-NMR spectra were analyzed using the ChenomX NMR Suite 7.1 software (Chenomx Inc., Edmonton, Alberta, Canada) for metabolite identification and quantification using the targeted profiling approach in the profiler module [[2](#_ENREF_2)]. Phasing was performed manually in the processor module of the ChenomX software, followed by deletion of the water region and baseline correction. All spectra were randomly ordered for targeted profiling to avoid progressive bias. DSS concentration was used as an internal reference to determine individual compound concentrations. All profiled metabolite chemical shift assignment were confirmed using the ^1^H-^13^C HSQC and TOCSY spectra and then compared with the Human Metabolome Database (version 2.5) [[4](#_ENREF_4)]. Compound concentrations were normalized to the sum of all concentration, excluding the three highest concentrated metabolites: glucose, lactate, and urea, which otherwise would dominate the normalization [[2](#_ENREF_2), [5](#_ENREF_5)]. It is important to note that lactate showed lower concentration in non-survivors vs. survivors (t-test <0.3). Obtained normalization data were then used for statistical analysis.

**GC-MS Spectrometry**

**Sample Preparation**

The chloroform–methanol–water extraction method (Bligh and Dyer) was used to remove proteins for metabolite extraction of 50µl plasma samples [[6](#_ENREF_6)]. The lower phase of chloroform and methanol was transferred to individual tubes and the upper phase, an aqueous layer was then dried using a SpeedVac (Eppendorf, Germany) and was used the next day for derivatization. For metabolite derivatization, 50 µl of 20 mg/ml methoxyamine-hydrochloride in pyridine solution was used for methoxyamination to preserve carbonyl groups. This step was followed by adding N-Methyl-N-(trimethylsilyl) trifluoroacetamide (MSTFA; Sigma-Aldrich, Germany) as a silylating agent. As a final step, samples were diluted with Hexane and were centrifuged at 20,844 x g for 5 minutes to remove any particles. For GC-MS analysis 200 µl of supernatant was used after transferring to a special GC-MS vial.

**Data Acquisition**

GC-MS analysis was performed using an Agilent chromatograph 7890A (Agilent Technologies, USA) coupled with a Waters GCT mass spectrometer, using a GC-TOF-MS technique. The mass spectrometer was programmed in the range of 50-800 *m/z*. Using Metabolite Detector software (Version 2.06, Technische Universität Carolo-Wilhelmina zu Braunschweig, Braunschweig, Germany), mass spectra were processed and analyzed to detect compounds. The GOLM metabolite database [[7](#_ENREF_7)] and NIST 2008 library [[8](#_ENREF_8)] were used to identify compounds.

**Metabolite Concentration Profiling**

GC-MS spectra were analyzed using the MetboliteDetector software package version 2.0. MetaboliteDetector is fully automated software used for deconvolution and analysis of raw GC-MS data, providing statistical analysis and visualization of identified and quantified metabolites. Raw GC-MS data files in NetCDF and FastFlight format were imported into the Metabolite Detector software for analysis using a scripted language C++ with a Qt-based graphical user interface. Metabolite Detector was used for: peak detection, chromatogram deconvolution, compound identification, compound integration and quantification and chromatogram alignment [[9](#_ENREF_9)].

**Data analysis**

NMR and GC-MS normalized data [[10](#_ENREF_10)] were log transformed, mean-centered or scaled to unit variance (UV) and then analyzed using the SIMCA-P+ program (Version 13.0, Umetrics AB, Umeå, Sweden).

**Verification of the OPLS-DA Model**

The quality of OPLS-DA data was verified by three performance indicators including: CV-ANOVA for assessing significance testing and reliability; R^2^Y, which describes the fraction of the variation of the Y (group status) variable explained by the non-orthogonal component model and indicates goodness of fit; and Q^2^Y, which is defined as the fraction of the variation of the same Y variable predicted by the model and indicates goodness of prediction [[11](#_ENREF_11)]. This predicted fraction of Y variable is obtained through an internal sevenfold cross validation by leaving one seventh (1/7^th^) of the samples out (jackknifing procedure) and then testing if the model will run with the remaining portion of data and will be predicted with 1/7^th^ removed. This step is repeated 7 times for each left out sample group. R^2^Y and Q^2^Y scores vary between 0 and 1, where R^2^Y and Q^2^Y scores close to 1 demonstrate an excellent model that has a high level of variance and a high reliability of the prediction. The R^2^Y score is always larger than the Q^2^Y score, and a difference of more than 0.3 between the R^2^ and Q^2^ scores needs to be examined carefully due to a probable external over fitting effect.

**Internal validation for prognosis of mortality in H1N1 pneumonia**

Internal validation was performed using cross validation (CV) and permutation test to estimate the predictive precision of OPLD-DA models. CV can measure the Q2Y value to show the predictive ability of OPLS-DA models by using leave out 1/7^th^ of samples as predictive set and calculate the Q2Y based on the rest of samples following repetition of CV analysis to leave out another 1/7^th^ of samples.

Internal validation was done for the 7 non-survivors and 14 age- and sex-matched survivors of H1N1 pneumonia patients. We also used internal validation for 7 non-survivors and 21 survivors’ cohort using those 20 metabolites and 63 features that were involved in the predictive OPLS-DA models to separate 7 non-survivors from 14 age- and sex-matched survivors.

Moreover, sensitivity and specificity and AUROC values were obtained using misclassification analysis by splitting the H1N1 samples (7 non-survivors and 14 survivors) and ventilated ICU controls into a training group and a prediction group and misclassification analysis was then performed on prediction group to measure sensitivity, specificity and AUROC.

**Variables important on projection (VIP)**

We performed VIP analyses for OPLS-DA models to select the metabolites/features that most significantly contributed to the separation of the two groups in a weighted fashion using a quantitative measure of discriminatory power of the metabolites that is ranked by a unitless number.

**Plasma Metabolomic Study of Patients**

Non-targeted plasma metabolic profiling was undertaken to explore differences in metabolite concentration between age- and sex-matched H1N1 pneumonia non-survivor and survivor plasma samples and between age and sex-matched H1N1 pneumonia patients and ICU ventilated controls patients and age-matched bacterial CAP patients; multivariate data analysis (MVDA) was carried out using SIMCA-P 13.0 software. The normalized data obtained from ^1^H-NMR and GC-MS spectra were first processed using the unsupervised method of PCA (Principal Component Analysis). This allowed simple and initial model creation to summarize the major sources of variation in the data matrix, identify outlier samples and explore the interval confidence [[12](#_ENREF_12)]. This was followed by orthogonal partial least-squares discrimination analysis (OPLS-DA) of the data to build prediction models.

**Metabolic Pathway Analysis**

A parallel pathway analysis of potential biomarkers was performed using Ingenuity Pathways Analysis (IPA) software (V3.1, Ingenuity Systems Inc., Mountain View, CA, USA) and an integrated software program named MetaboAnalyst; a free web-based tool that combines results from powerful pathway enrichment analysis involved in the conditions under study. Both tools provide comparable features of biochemical pathways using database sources including the KEGG (http://www.genome.jp/kegg/), Human Metabolome Database (http://www.hmdb.ca/), SMPD (http://www.smpdb.ca/) and METLIN (http://metlin.scripps.edu/) to identify the affected metabolic pathways and allow visualization of the interactions (see the Tables E1-E4 for pathways analysis results and figures E3A, E3B, E4A and E4B for graphically displayed results).

**Further Discussion about Pathway Analysis**

**Prognosis of mortality in H1N1 pneumoniae**

Analysis of the potential pathways involved in differentiating H1N1 survivors from non-survivors is further explored in addition to that described in the manuscript. If we first look at energy metabolism we see the importance of glutamate as an involved metabolite. Glutamate is known as a supplier of the majority of carbon, nitrogen, and energy for cell growth and division. Several biological pathways are connected to this metabolic pathway, including the urea cycle and glycolysis. In animals or humans, glutamate plays a major role in amino acid interconversion [[13](#_ENREF_13)]. Increased plasma levels of glutamate could be closely related to protein breakdown and, in addition to the imbalance of other amino acids, is associated with sepsis mortality [[14](#_ENREF_14)] as it is in this study. Interestingly, a glutamine supplementing diet has been shown to be part of an effective therapy to decrease infections in surgical patients [[15](#_ENREF_15), [16](#_ENREF_16)].

Again, focusing on energy metabolism, pyruvate metabolism (an important pathway identified in this study, Table 2) is also involved in alanine and glutamate metabolism regulation. Pyruvate is an important energy-related metabolite in both hypoxic and non-hypoxic conditions. In non-hypoxic conditions when glycolysis occurs, it is converted into abundant energy through the TCA cycle. In hypoxic conditions, pyruvate is converted to lactate with much less energy production. Elevated lactate is extremely common in all forms of sepsis even when hypoxia does not appear to be present. This may be because pyruvate itself may accumulate in sepsis when glycolytic pathways are over-activated producing pyruvate even more so than lactate. Based on this theory, lactate is a byproduct of the overall accelerated glycolysis, not due to tissue hypoxia per se. In fact, some have hypothesized that lactate production may be physiologically beneficial in septic patients where the acidic environment may aid in tissue oxygen delivery [[17](#_ENREF_17)]. The decrease of lactate in H1N1 non-survivors compared with survivors suggests this phenomenon.

In further consideration of the involvement of energy metabolism in differentiating survivors from non-survivors, one must consider the involvement of alanine metabolism (as shown in table 2). Alanine plays an important role in transporting metabolic compounds for amino groups other than glutamine. Thus, our data suggests that pyruvate and alanine metabolism may play significant roles along with glutamate metabolism in surviving H1N1 pneumonia.

Interesting and potentially very informative, the increases of two TCA cycle intermediates, 2-oxoglutarate and fumarate, were observed to be elevated in pneumococcal pneumonia infection [[18](#_ENREF_18)], whereas they were shown to decrease in non-survivor vs. survivor of H1N1 pneumonia patients. It is unclear whether these biomarkers belong to the pneumococcal infection metabolomics profile itself or are driven mainly by a general stress response of the body. Whatever is the case; this shows a potential significant difference between pneumococcal pneumonia metabolomics and H1N1 pneumonia metabolomics.

Another highlight of the importance of energy metabolism is the involvement of glucogenic pathway amino acids. It is noteworthy that all the amino acids shown in table 2 belong to non-essential amino acids that take part in many metabolic pathways and biological processes. One important pathway that these amino acids contribute to is the gluconeogenesis pathway, hence these amino acids are known as glucogenic amino acids. These pathways attempt to restore glucose appropriate levels as glucose is needed for energy. When glucose is not available, ketone bodies are produced in liver mitochondria. In a related fashion, tyrosine and phenylalanine are ketogenic as their degradation yields ketone bodies [[19](#_ENREF_19)]. There is at least some evidence that tight glucose control in critically ill patients, using insulin which makes intracellular glucose more available, has been shown to have a superior outcome compared to those where tight glucose control was not used [[20](#_ENREF_20), [21](#_ENREF_21)]

Metabolomic pathway analysis reveals that loss of protein synthesis is an important feature in the non-survivors in H1N1 pneumonia. tRNA charging pathway (Table 2) is one of the suggested pathways involved in H1N1 pneumonia patients by IPA analysis. This suggests that protein biosynthesis perturbation is occurring in H1N1 infection. The tRNA charging pathway perturbation could be caused by reducing amino acid availability leading to the accumulation of non-aminoacetylated tRNAs and a disruption in protein biosynthesis. Also, a decrease in ATP molecules due to a loss of glucogenesis can subsequently cause a decrease in AMP thereby causing a decrease of protein synthesis seen when proteins are consumed as an energy source of the living cells. Changing β-alanine metabolism and tyrosine degradation (Table 2) could also result in deamination and decarboxylation of amino acids and subsequently loss of protein biosynthesis. All of these changes result in decreased protein synthesis especially noted in the non-survivors.

One important feature of the survivors was the presence of fever. There were no statistically significant differences between survivors and non-survivors for demographic variables, clinical findings, and comorbidities with the exception of fever > 38.0 which was significantly more often observed in survivors (Table S15). The capacity to generate a fever may be reflected in those that have a robust immune response vs. those that may have a more sluggish immune capacity. In addition, if energy metabolism is disrupted it may inhibit the ability to generate a fever. Thus, the survivors, those that can generate a fever, may reflect the ability to utilize or generate energy and protein to retain an immune capacity. This hypothesis and the ability to generate fever in sepsis survivors has been described previously[[22](#_ENREF_22), [23](#_ENREF_23)].

**Further Discussion about Diagnostic Pathway Analysis**

**Diagnosis of H1N1 patients from ICU ventilated controls**

Taurine and hypotaurine metabolism showed high pathway impact in pathway analysis for diagnosis of H1N1 patients from ICU ventilated controls. Taurine is an important compound in bile acid conjugation in the liver suggesting that involvement of the liver during the H1N1 infection can affect its metabolism. Moreover, Taurine is an important intracellular free amino acid that is known as an antioxidant and a neuromodulator and is also involved in regulation of osmolarity in the neural retina and brain. H1N1 patients showed lower concentration of taurine compared to ICU ventilated controls [[24-26](#_ENREF_24)].

Decreased serine and glycine in H1N1 patients compared to ICU ventilated controls may be associated with mitochondria dysfunction in the H1N1 infection where the glycine derived enzymes are involved in oxidation and decarboxylation of serine. Also, threonine is converted to glycine and then serine to produce more pyruvate, acetaldehyde and acetate as precursors of acetyl-CoA. The glycine, serine and threonine metabolism is the biological pathway that is more active in ICU ventilated controls than H1N1 patients. Glutamate, alanine and asparate (glucogenic amino acids) are connected to citrate cycle or to pyruvate via transamination. The metabolic pathways of these amino acids and citrate cycle are more active biological pathways in ICU ventilated controls compared to H1N1 patients.

Increased levels of phenylalanine and pyruvate in H1N1 patients may be associate with increased protein catabolism [[19](#_ENREF_19)]. Moreover, the increased level of beta-alanine in H1N1 patients could indicate catabolism of bases when the pyrimidine nucleotides and nucleosides are degraded. In a general degradation pathway, two base residues uracil and thymine undergo deamination and decarboxylation to produce β-alanine and 3-aminoisobutyrate.

Arginine itself is involved in several biological mechanisms and increased level of arginine in H1N1 patients could indicate arginine has not been used for synthesis of polyamines that are important compounds for cellular activities such cell growth, DNA synthesis and cell membrane transportation. Methane metabolism is another pathway that has been activated in H1N1 as compared to ICU ventilated controls. This could result in increased production of CO_2_ when the gas exchange is interrupted and methogenesis occurs due to CO_2_ reduction. Glyoxylate metabolism in H1N1 compared to ICU ventilated controls indicates the conversion of acetyl-CoA into succinate and malate (citrate cycle dicarboxylate) for gluconeogensis, as well, succinate is transported to the mitochondrion for respiration [[27](#_ENREF_27)]. Also, phenylalanine can participate in glucogensis showing plasma increased level in H1N1 patients.

Changes (decreases) in fatty acids in H1N1 patients compared to ICU ventilated controls could indicate the robust metabolic alteration in cellular processes. Viral infection can be involved in the demolition of all fundamental cellular activities where the glycerolipid and other fatty acids are requirements for cellular process. On the other hand, it is possible that the decreased levels of fatty acids in H1N1 patients originates from pyruvate and acetyl-CoA metabolism that are directly involved in fatty acids biosynthesis and metabolism [[28](#_ENREF_28)]. In summary, the metabolic pathways that appear to differentiate H1N1 pneumonia from the ICU controls appear to involve the changes in galactose, glucose, lactic acid, pyroglutamic acid, mannose, urea, valine, octadecadienoic acid, phosphoric acid, octadecanoic acid that are detected by GC-MS analysis for the diagnosis of H1N1 from ICU ventilated controls. Dimethylamine, β-alanine, asparate, phenylalanine, formate, isopropanol, citrate, taurine, glycine, 2-oxoglutarate and glutamine are the most important metabolites detected by NMR that separate H1N1 and ICU controls.

**Supplement References**

1. Kellum JA, Kong L, Fink MP, Weissfeld LA, Yealy DM, Pinsky MR, Fine J, Krichevsky A, Delude RL, Angus DC: **Understanding the inflammatory cytokine response in pneumonia and sepsis: results of the Genetic and Inflammatory Markers of Sepsis (GenIMS) Study**. *Archives of internal medicine* 2007, **167**(15):1655-1663.

2. Weljie AM, Newton J, Mercier P, Carlson E, Slupsky CM: **Targeted profiling: quantitative analysis of 1H NMR metabolomics data**. *Anal Chem* 2006, **78**(13):4430-4442.

3. Nicholson JK, Foxall PJ, Spraul M, Farrant RD, Lindon JC: **750 MHz 1H and 1H-13C NMR spectroscopy of human blood plasma**. *Anal Chem* 1995, **67**(5):793-811.

4. Wishart DS, Knox C, Guo AC, Eisner R, Young N, Gautam B, Hau DD, Psychogios N, Dong E, Bouatra S *et al*: **HMDB: a knowledgebase for the human metabolome**. *Nucleic Acids Research* 2009, **37**(Database):D603-D610.

5. Dieterle F, Ross A, Schlotterbeck G, Senn H: **Probabilistic quotient normalization as robust method to account for dilution of complex biological mixtures. Application in 1H NMR metabonomics**. *Anal Chem* 2006, **78**(13):4281-4290.

6. Bligh EG, Dyer WJ: **A rapid method of total lipid extraction and purification**. *Can J Biochem Physiol* 1959, **37**(8):911-917.

7. Hummel J SJ, Walther D, Kopka J: **The Golm Metabolome Database: a database for GC-MS based metabolite profiling. Metabolomics**, vol. 18: Springer Berlin / Heidelberg; 2007.

8. Stein SE: **Chemical substructure identification by mass spectral library searching**. *Journal of the American Society for Mass Spectrometry* 1995, **6**(8):644-655.

9. Hiller K, Hangebrauk J Fau - Jager C, Jager C Fau - Spura J, Spura J Fau - Schreiber K, Schreiber K Fau - Schomburg D, Schomburg D: **MetaboliteDetector: comprehensive analysis tool for targeted and nontargeted GC/MS based metabolome analysis**. *Anal Chem* 2009, **81**(9):3429-3439.

10. Farshidfar F, Weljie AM, Kopciuk K, Buie WD, Maclean A, Dixon E, Sutherland FR, Molckovsky A, Vogel HJ, Bathe OF: **Serum metabolomic profile as a means to distinguish stage of colorectal cancer**. *Genome Med* 2012, **4**(5):42.

11. Holmes E, Loo R, Stamler J, Bictash M, Yap I, Chan Q, Ebbels T, De Iorio M, Brown I, Veselkov K *et al*: **Human metabolic phenotype diversity and its association with diet and blood pressure**. 2008(1476-4687 (Electronic)).

12. Eriksson L, Johansson E, Kettaneh-Wold N, Trygg J, Wikström C, S W: **Multi- and Megavariate Data Analysis Part I: Basic Principles and Applications**, vol. 1. Umeå: Umetrics AB, Sweden; 2004.

13. Brosnan JT: **Glutamate, at the interface between amino acid and carbohydrate metabolism**. *J Nutr* 2000, **130**(4S Suppl):988S-990S.

14. Poeze M, Luiking YC, Breedveld P, Manders S, Deutz NE: **Decreased plasma glutamate in early phases of septic shock with acute liver dysfunction is an independent predictor of survival**. *Clin Nutr* 2008, **27**(4):523-530.

15. Hirose T, Shimizu K, Ogura H, Tasaki O, Hamasaki T, Yamano S, Ohnishi M, Kuwagata Y, Shimazu T: **Altered balance of the aminogram in patients with sepsis - The relation to mortality**. *Clin Nutr* 2014, **33**(1):179-182.

16. Cohen J, Chin w D: **Nutrition and sepsis**. *World Rev Nutr Diet* 2013, **105**:116-125.

17. Gore DC, Jahoor F Fau - Hibbert JM, Hibbert Jm Fau - DeMaria EJ, DeMaria EJ: **Lactic acidosis during sepsis is related to increased pyruvate production, not deficits in tissue oxygen availability**. 1996(0003-4932 (Print)).

18. Slupsky CM: **Nuclear magnetic resonance-based analysis of urine for the rapid etiological diagnosis of pneumonia**. *Expert Opinion on Medical Diagnostics* 2011, **5**(1):63-73.

19. Michal G, Schomburg, D. (ed.): **Biochemical Pathways: an atlas of biochemistry and molecular biology**. Hoboken, New Jersey: John Wiley and Sons; 2012.

20. Van den Berghe G, Wilmer A, Hermans G, Meersseman W, Wouters PJ, Milants I, Van Wijngaerden E, Bobbaers H, Bouillon R: **Intensive insulin therapy in the medical ICU**. *N Engl J Med* 2006, **354**(5):449-461.

21. van den Berghe G, Wouters P, Weekers F, Verwaest C, Bruyninckx F, Schetz M, Vlasselaers D, Ferdinande P, Lauwers P, Bouillon R: **Intensive insulin therapy in critically ill patients**. *N Engl J Med* 2001, **345**(19):1359-1367.

22. Langley RJ, Tsalik EL, van Velkinburgh JC, Glickman SW, Rice BJ, Wang C, Chen B, Carin L, Suarez A, Mohney RP *et al*: **An integrated clinico-metabolomic model improves prediction of death in sepsis**. *Sci Transl Med* 2013, **5**(195):195ra195.

23. Glickman SW, Cairns CB, Otero RM, Woods CW, Tsalik EL, Langley RJ, van Velkinburgh JC, Park LP, Glickman LT, Fowler VG, Jr. *et al*: **Disease progression in hemodynamically stable patients presenting to the emergency department with sepsis**. *Acad Emerg Med* 2010, **17**(4):383-390.

24. Huxtable RJ: **Taurine in the central nervous system and the mammalian actions of taurine**. *Prog Neurobiol* 1989, **32**(6):471-533.

25. Wu J-Y, Chen W, Tang X, Jin H, Foos T, Schloss J, Davis K, Faiman M, Hsu C-C: **Mode of Action of Taurine and Regulation Dynamics of Its Synthesis in the CNS**. *Advances in Experimental Medicine and Biology* 2002, **483**:35-44.

26. Rosenthal MD, Glew RH: **Medical Biochemistry: Human Metabolism in Health and Disease**: Wiley; 2009.

27. Villas-Boas SG, Kesson M, Nielsen J: **Biosynthesis of glyoxylate from glycine in Saccharomyces cerevisiae**. *FEMS Yeast Res* 2005, **5**(8):703-709.

28. Henry SA, Kohlwein SD, Carman GM: **Metabolism and regulation of glycerolipids in the yeast Saccharomyces cerevisiae**. *Genetics* 2012, **190**(2):317-349.
