# Supplementary material for: Plasma metabolomics for the diagnosis and prognosis of H1N1 influenza pneumonia
Source: Crit Care. 2017 Apr 19;21:97. doi: 10.1186/s13054-017-1672-7 (PMC5397800; doi:10.1186/s13054-017-1672-7)
Supplement: Supplementary file 2 — Supplementary material, figures, and tables. (DOCX 1.79 mb) [file 13054_2017_1672_MOESM2_ESM.docx]

**Supplement Figure and Table**

**Figure S1**. The unsupervised PCA analysis of plasma from all H1N1 patients vs. positive bacterial culture CAP patients shows general separation (clustering). **A:** PCA plot for NMR data, **B;** PCA plot for GC-MS data. The X axis is first principal component that has the greatest variation between the samples and the Y axis is the second principal component that has the second greatest variation between the samples.


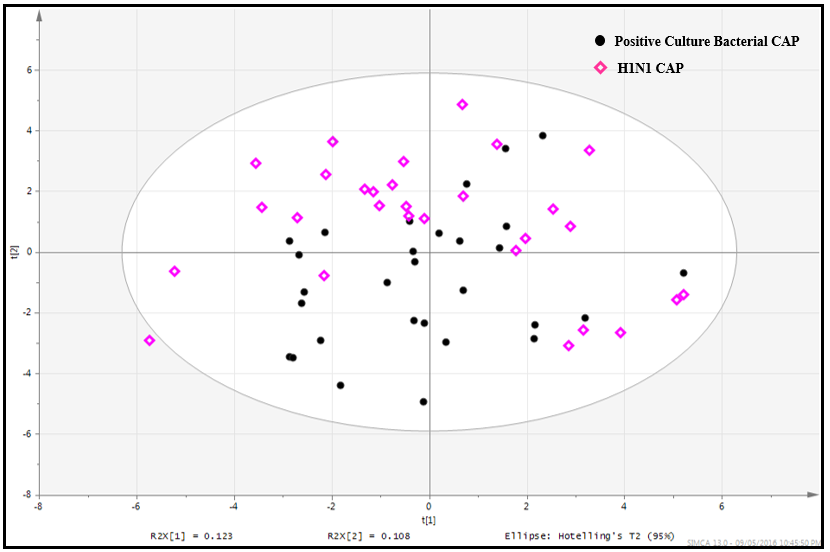


**Figure S1A**


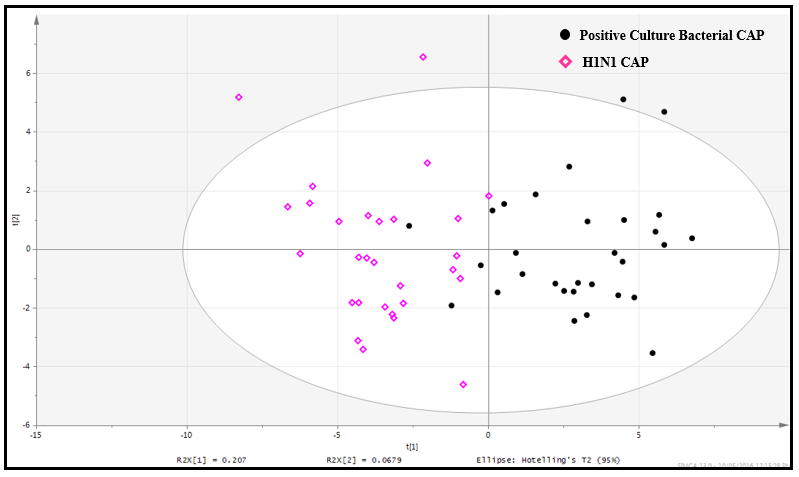


**Figure S1B**

**Figure S2**. The unsupervised PCA analysis of plasma from all H1N1 patients vs. ICU ventilated controls shows general separation (clustering) and reveals the data outliers. **A:** PCA plot for NMR data, **B:** PCA plot for GC-MS data. The X axis is first principal component that has the greatest variation between the samples and the Y axis is the second principal component that has the second greatest variation between the samples.


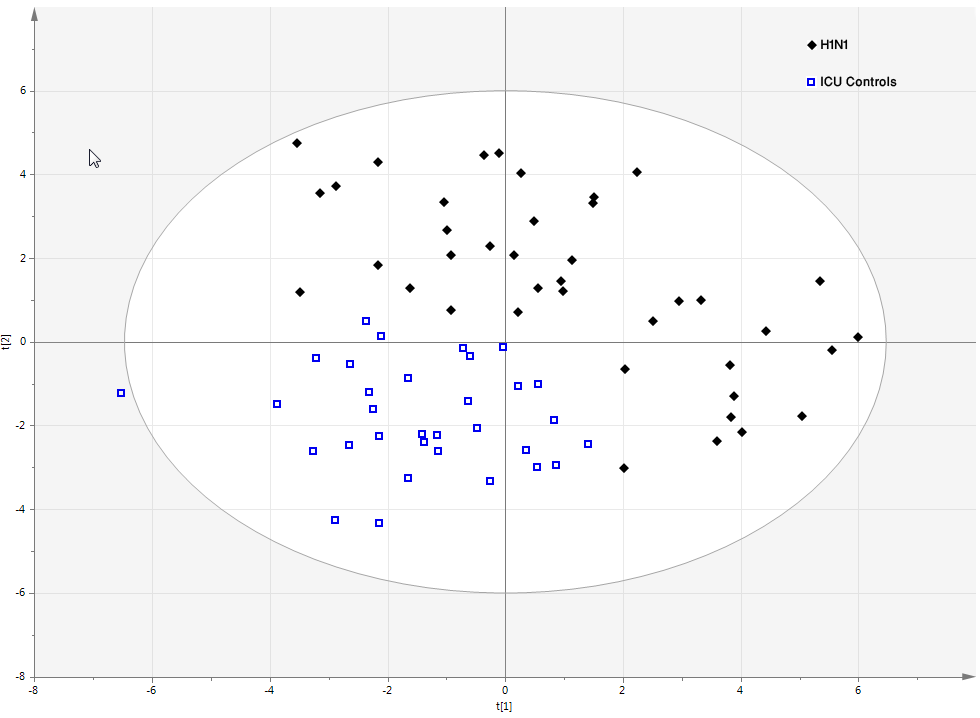


**Figure S2A**


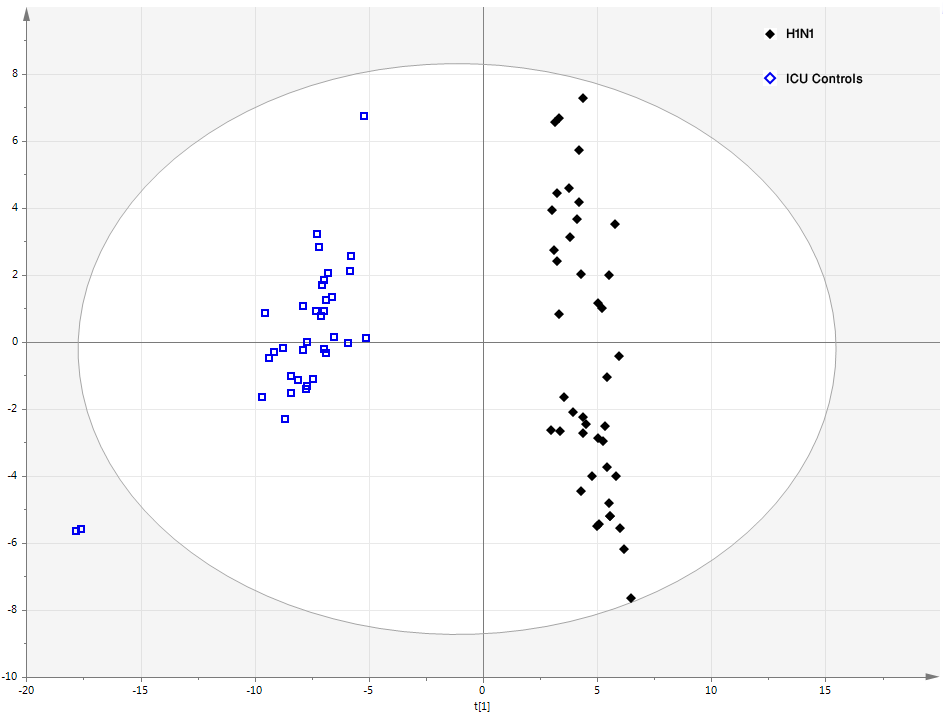


**Figure S2B**

**Figure S3**. The unsupervised PCA analysis of plasma from H1N1 survivors vs. non-survivors shows general separation (clustering) and reveals the data outliers. **A:** 2-dimensional PCA plot for NMR data, **B:** a 3-dimentional PCA plot for GC-MS data. The X axis is first principal component that has the greatest variation between the samples, the Y axis is the second principal component that has the second greatest variation between the samples and the Z axis is the third principal component that has the third greatest variation between the samples.


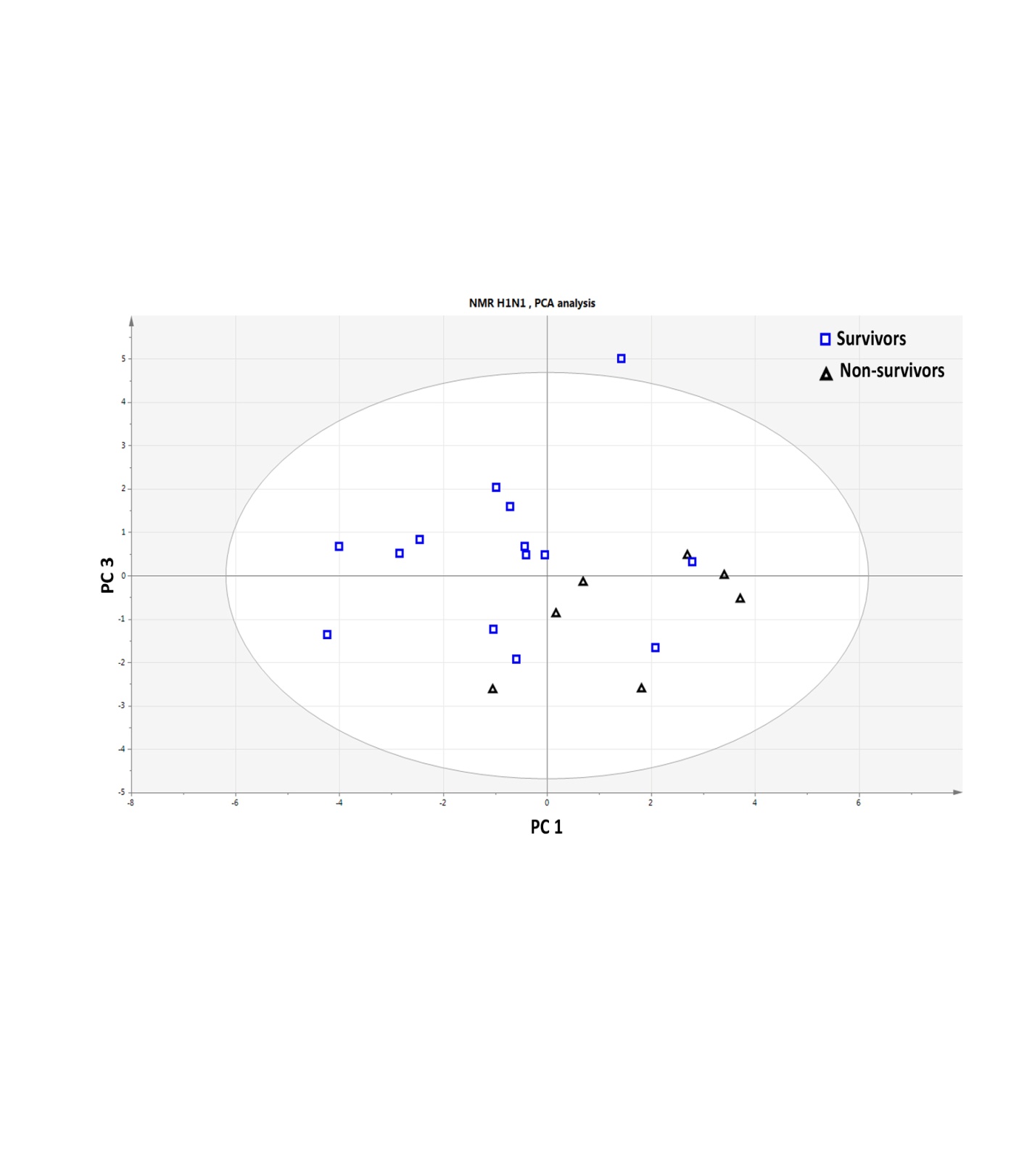


**Figure S3A**


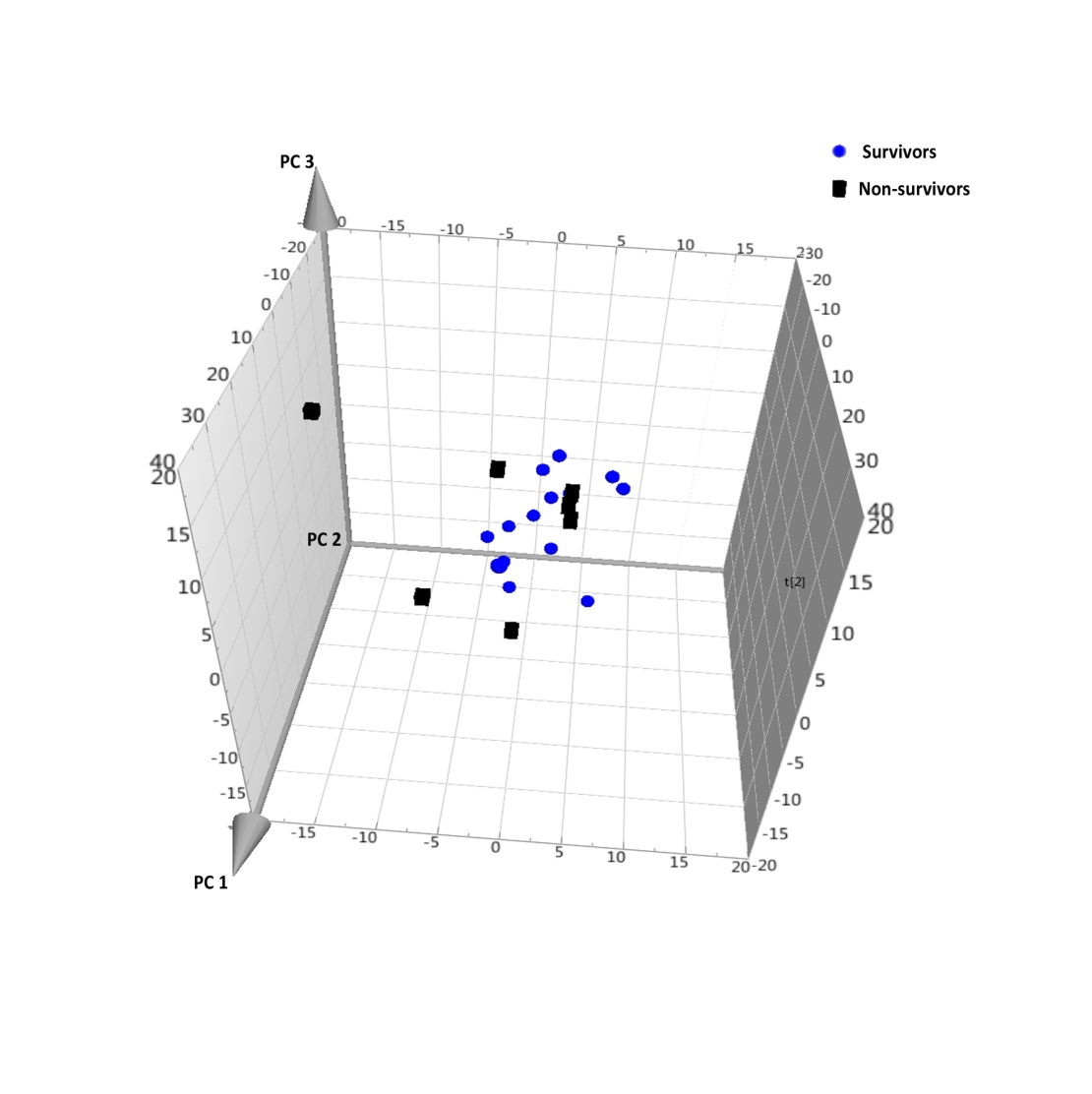


**Figure S3B**

**Figure S4A**.The supervised O2PLS analysis shows the discrimination between male and female samples but we still observe the separation between non-survivors and survivors using NMR data. (R^2^Y= 0.714, Q^2^Y=0.516)


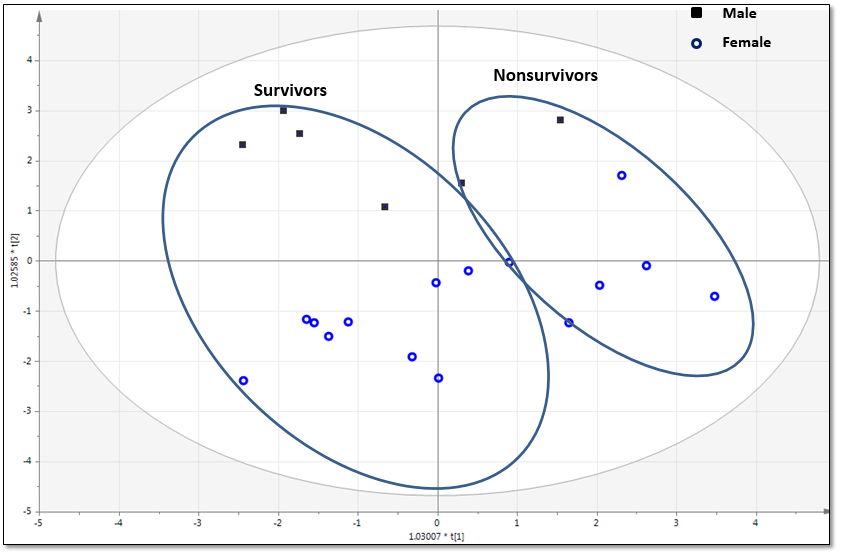


**Figure S4A**

**Figure S4B**.The supervised O2PLS analysis shows the discrimination between age > 40 and age < 40 but we still observe the separation between non-survivors and survivors using NMR data. (R^2^Y= 0.64, Q^2^Y=0.418).


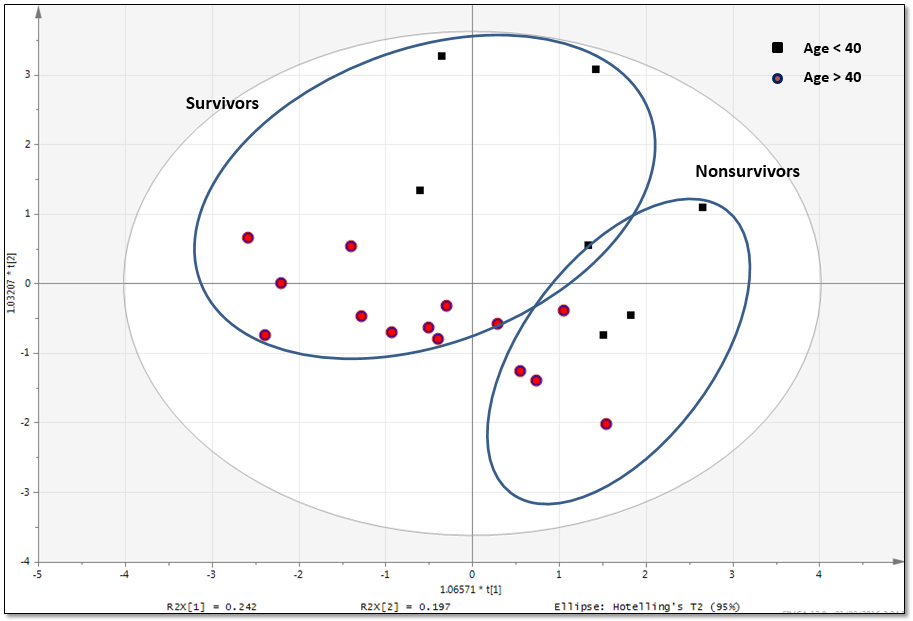


**Figure S4B**

**Figure S4C**.The supervised O2PLS analysis shows the discrimination between BMI > 35 and BMI < 35 but we still observe the separation between non-survivors and survivors using NMR data. (R^2^Y= 0.475, Q^2^Y=0.464).


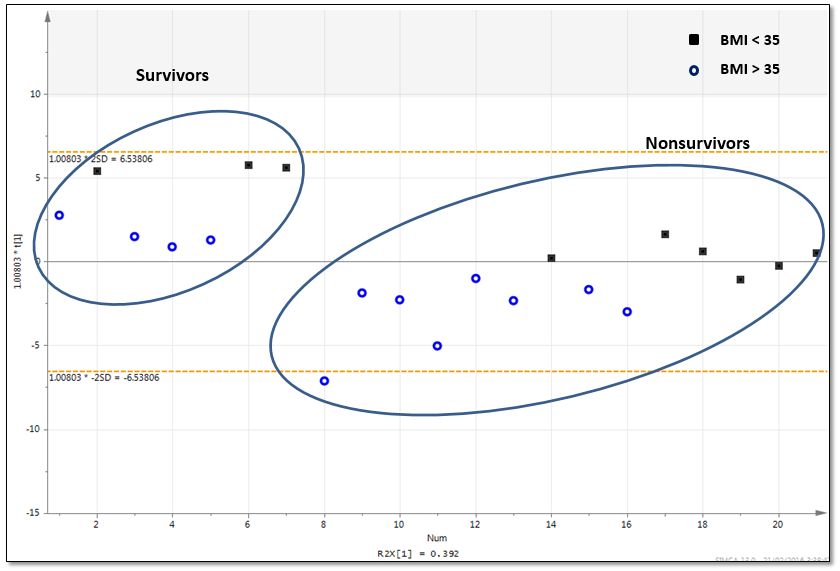


**Figure S4C**

**Figure S5.** Coefficient plot for the diagnosis of H1N1 compared positive bacterial culture CAP sample. **A:** Coefficient plot of NMR data, **B:** Coefficient plot of GC-MS data. The metabolites showing the most significant differentiation between H1N1 and bacterial culture CAP sample are shown.


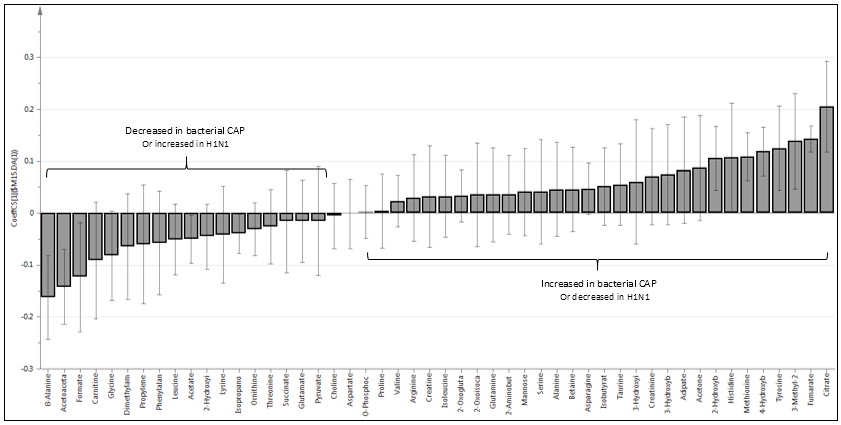


**Figure S5A**


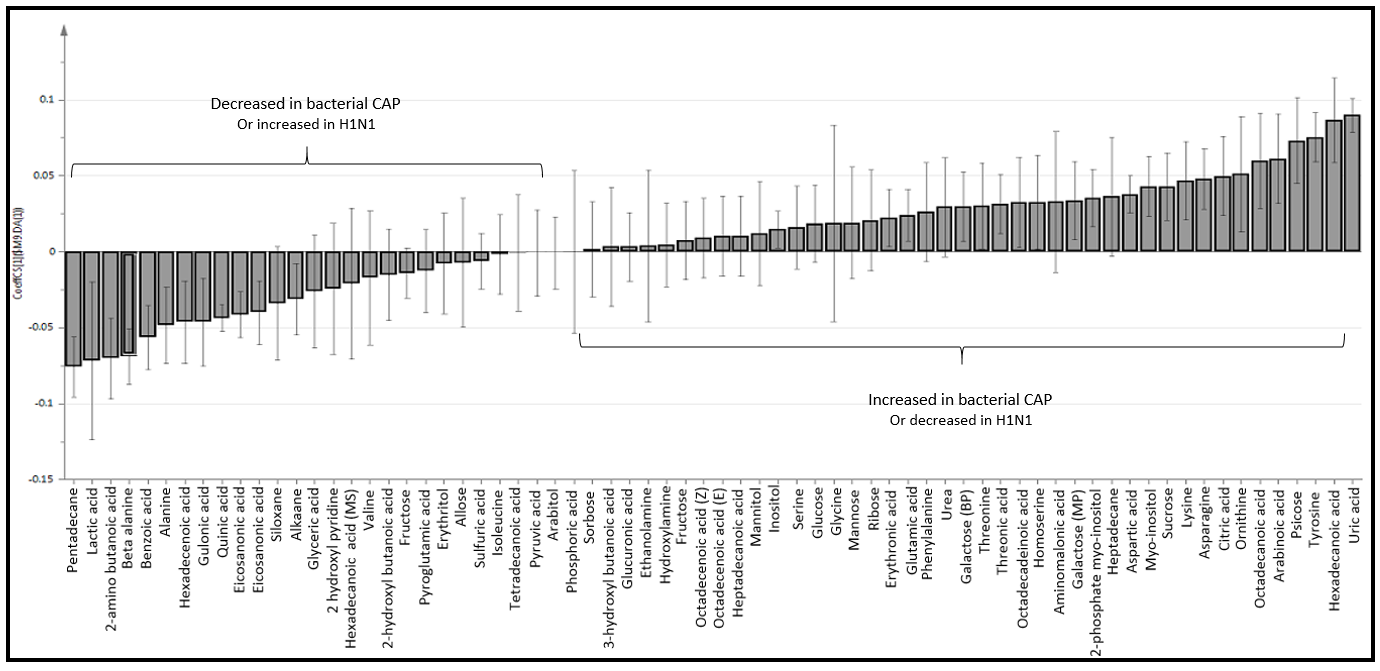


**Figure S5B**

**Figure S6.** S-plot analysis to identify putative plasma biomarkers for diagnosis H1N1 pneumonia vs. positive bacterial culture CAP patients. X-axis shows the variable magnitude (modelled co-variation), the magnitude increases to the sides from the center. Y-axis shows the reliability of variable (modelled correlation), the reliability increases as one moves down and up from the center. **A:** S-plot of NMR data, **B:** S-plot of GC-MS data. Individual metabolites are labeled.


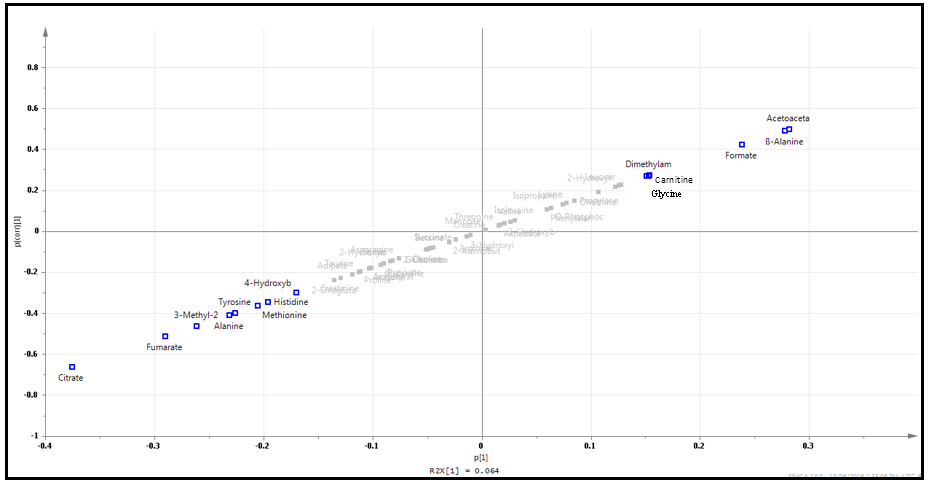


**Figure S6A**


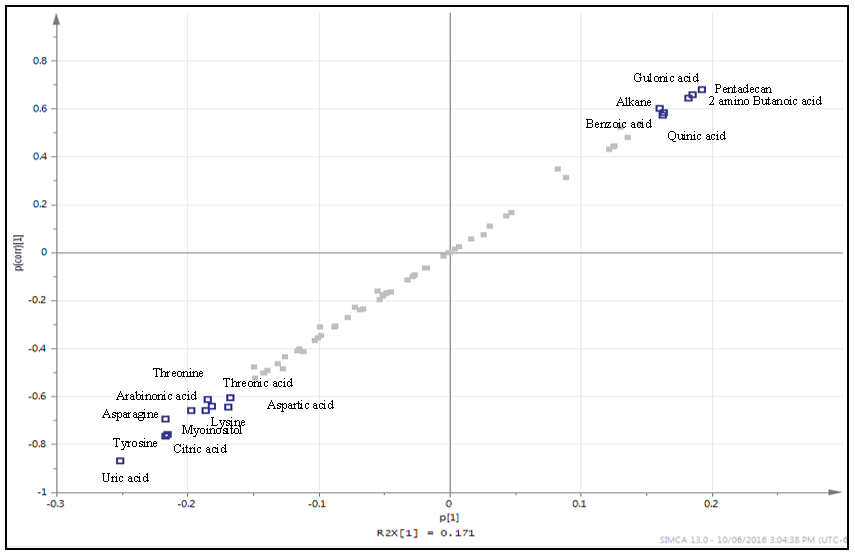


**Figure S6B**

**Figure S7.** Coefficient plot of the plasma metabolites for the diagnosis of H1N1 compared ICU controls. **A:** Coefficient plot of NMR data, **B:** Coefficient plot of GC-MS data. The metabolites showing the most significant differentiation between H1N1 and ICU controls are shown.


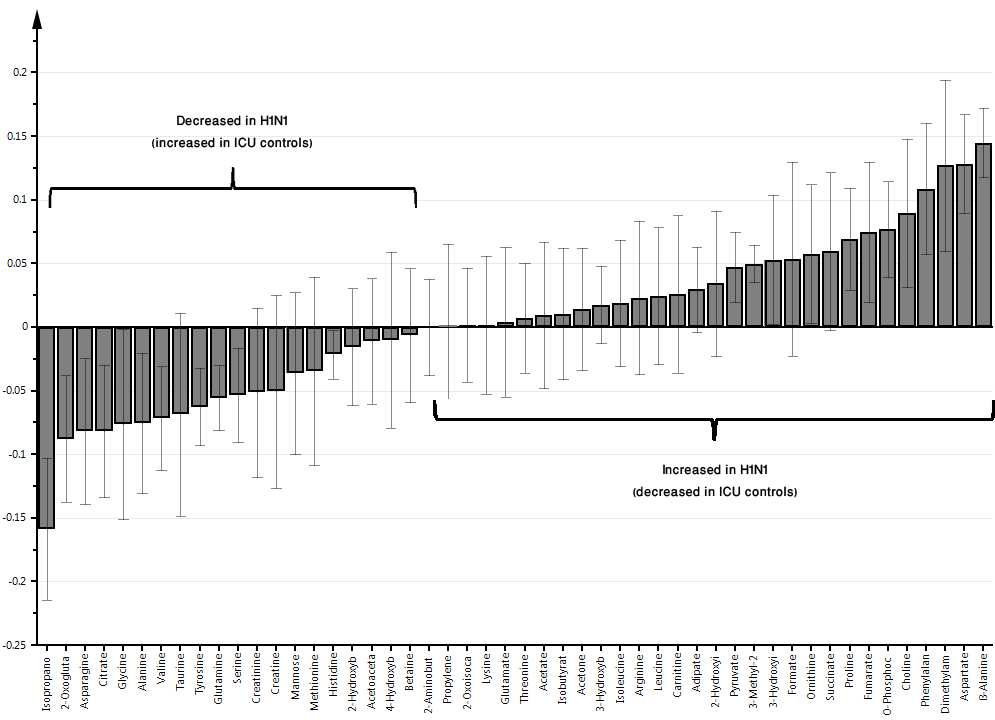


**Figure S7A**

**
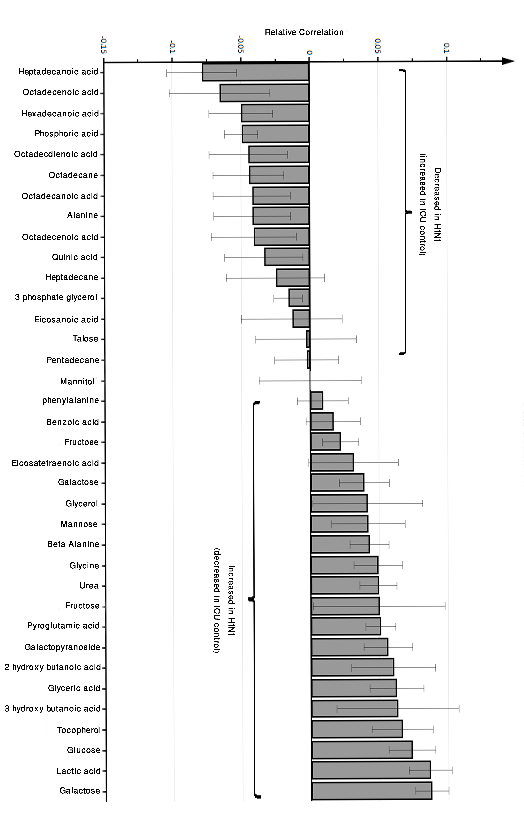
**

**Figure S7B**

**Figure S8.** S-plot analysis to identify putative biomarkers for diagnosis H1N1 pneumonia vs ventilated ICU controls. X-axis shows the variable magnitude (modelled co-variation), the magnitude increase to the sides from center. Y-axis shows the reliability of variable (modelled correlation), the reliability increases toward to down and up from the center. **A:** S-plot of NMR data, **B:** S-plot of GC-MS data.


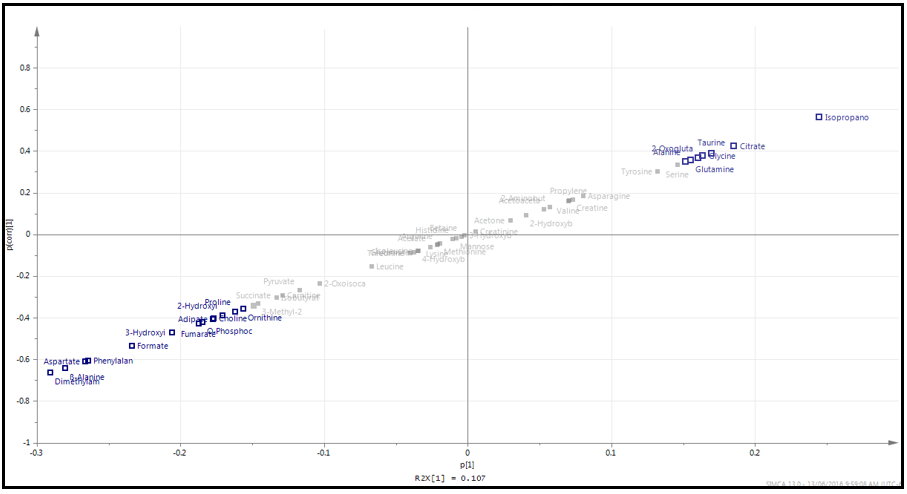


**Figure S 8A**

**
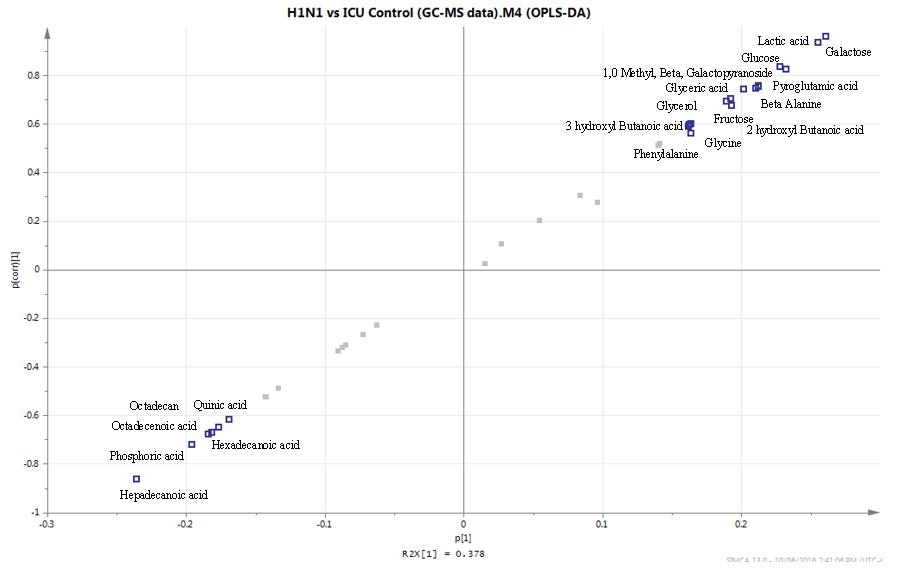
**

**Figure S 8B**

**Figure S 9.** Coefficient plot refers to scaled and centered metabolites’ relative correlation data, with the 95% confidence interval derived from jack-knifing for prognosis of mortality. A: Coefficient plot of NMR data, B: Coefficient plot of GC-MS data. The plasma metabolites showing the most significant differentiation between survivors and non-survivors are shown.


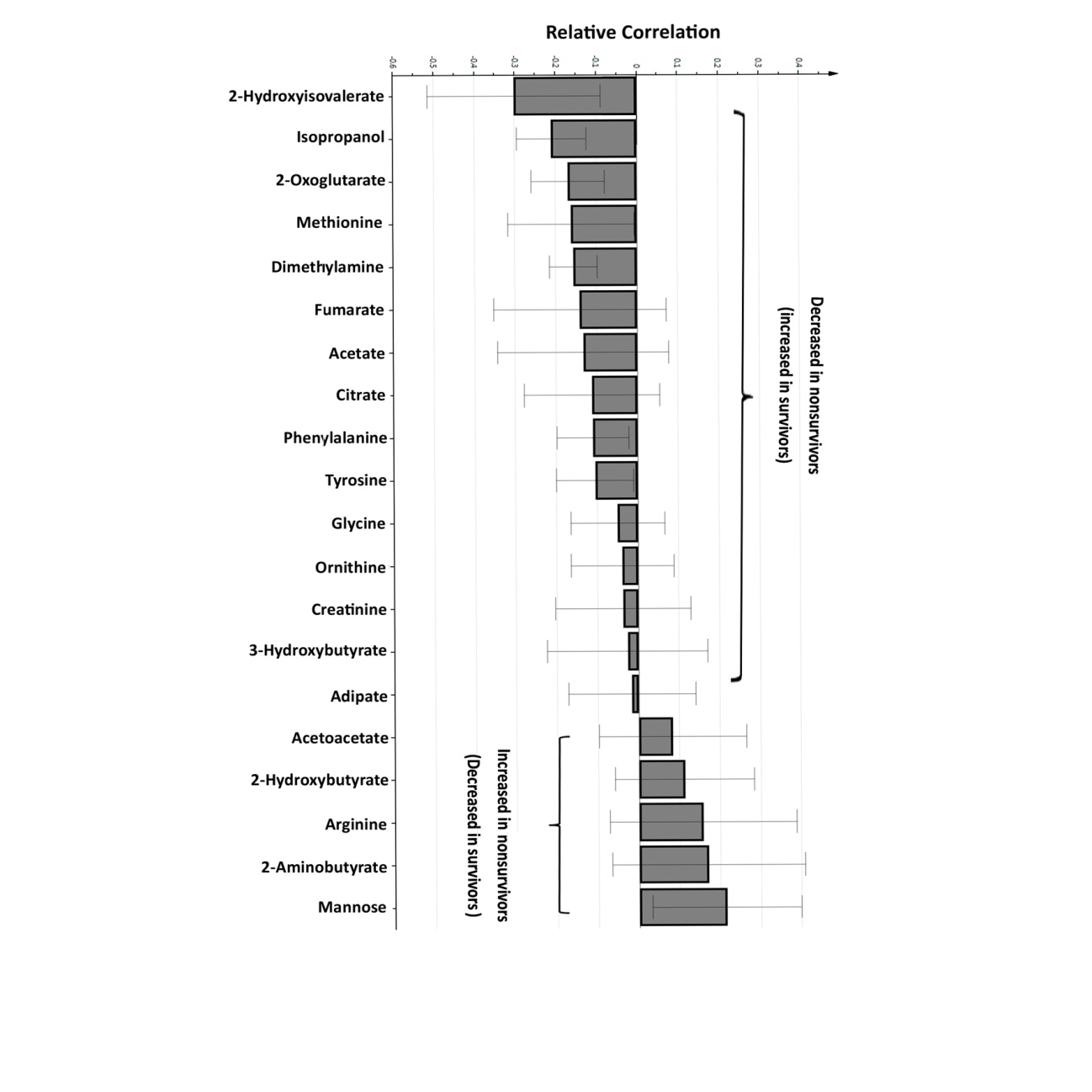

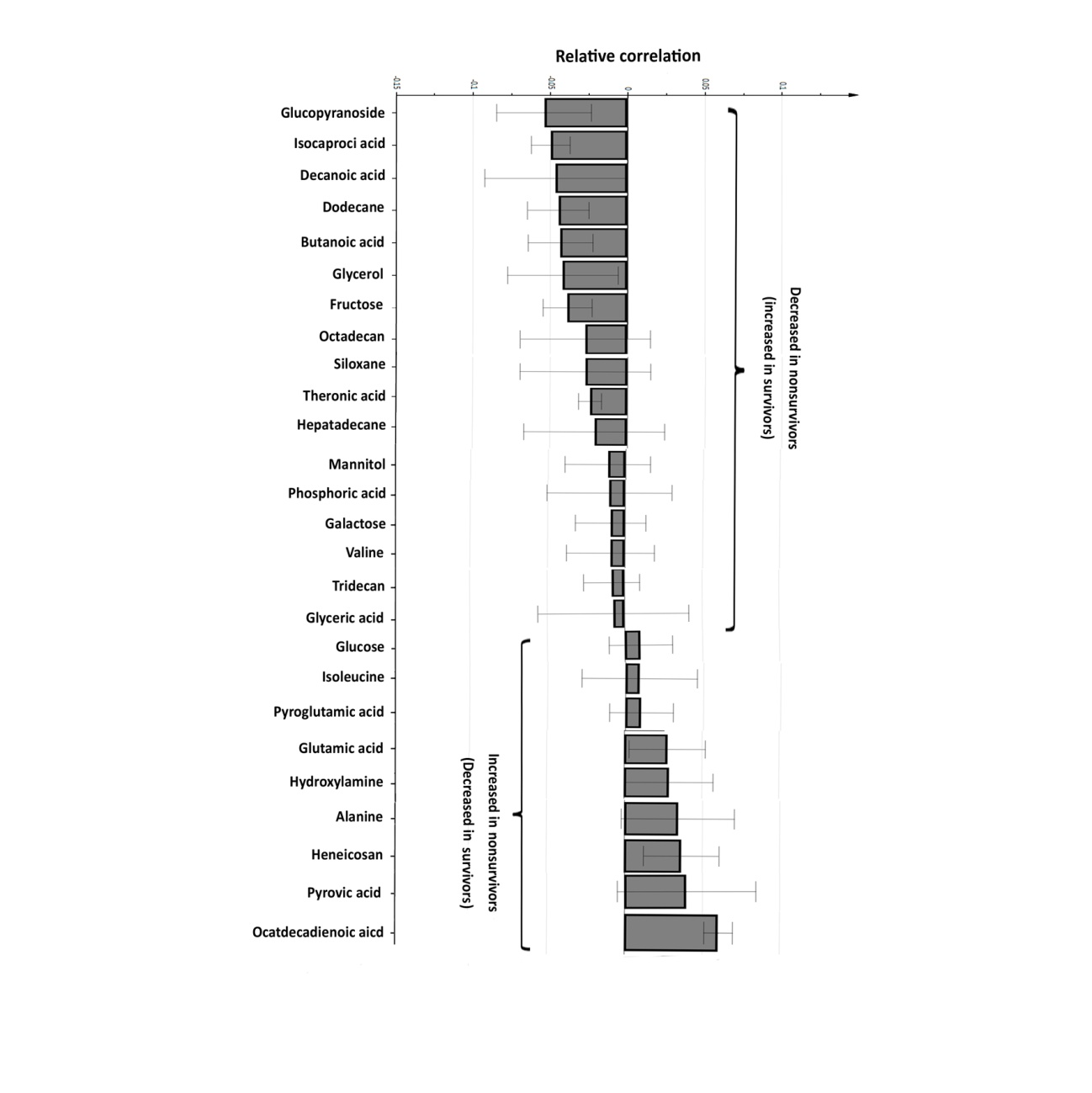


**Figure S9A** **Figure S9B**

**Figure S10.** S-plot analysis to identify putative biomarkers for prognosis of H1N1 nonsurvivors vs. H1N1 survivors. X-axis shows the variable magnitude (modelled co-variation), the magnitude increase to the sides from center. Y-axis shows the reliability of variable (modelled correlation), the reliability increases toward to down and up from the center. **A:** S-plot of NMR data, **B:** S-plot of GC-MS data.


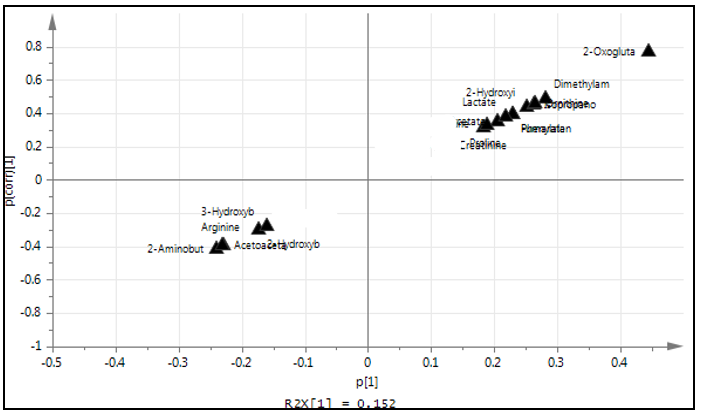


**Figure S10A**


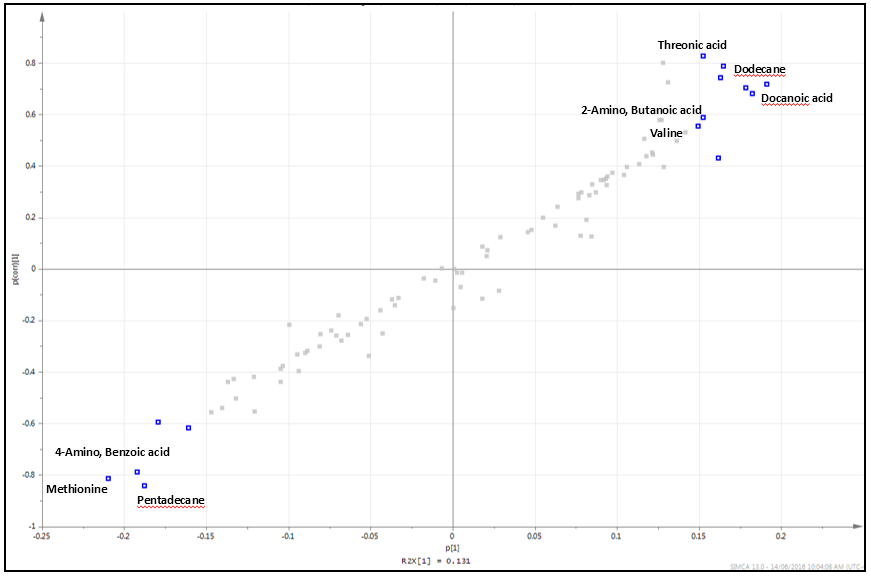


**Figure S10B**

**Tables S1 and S2**. Detailed results from the univariate t-test analysis to determine significant differences of plasma metabolites/features between H1N1 and positive bacterial culture patients using MetaboAnalyst software. **Table S1:** NMR data. **Table S2:** GC-MS data.

**Table S1**

| Name | Mean (SD) of Positive Bacterial Culture | Mean (SD) of H1N1 | p-value | Change in Positive Bacterial Culture/H1N1 |
| --- | --- | --- | --- | --- |
| Fumarate | 0.439 (0.881) | -0.439 (0.927) | 0.0005 | Up |
| Formate | -0.360 (0.868) | 0.360 (1.007) | 0.0051 | Down |
| Tyrosine | 0.354 (0.675) | -0.354 (1.149) | 0.0064 | Up |
| Alanine | 0.314 (0.994) | -0.314 (0.918) | 0.0154 | Up |
| 4-Hydroxybutyrate | 0.292 (0.864) | -0.292 (1.055) | 0.0247 | Up |
| Acetoacetate | -0.488 (0.866) | 0.488 (0.890) | < 0.0001 | Down |
| Citrate | 0.606 (0.831) | -0.606 (0.764) | < 0.0001 (W) | Up |
| 3-Methyl-2-oxovalerate | 0.426 (0.972) | -0.426 (0.846) | 0.0001 (W) | Up |
| Histidine | 0.349 (0.841) | -0.349 (1.037) | 0.0006 (W) | Up |
| Beta-Alanine | -0.426 (0.574) | 0.426 (1.153) | 0.0016 (W) | Down |
| Adipate | 0.256 (0.633) | -0.256 (1.224) | 0.0087 (W) | Up |
| Carnitine | -0.226 (0.793) | 0.226 (1.141) | 0.0181 (W) | Down |
| Leucine | -0.260 (1.019) | 0.260 (0.926) | 0.0254 (W) | Down |
| Methionine | 0.346 (1.110) | -0.346 (0.745) | 0.0311 (W) | Up |

**Table S2**

| Name | Mean (SD) of Positive Bacterial Culture | Mean (SD) of H1N1 | p-value | Change in Positive Bacterial Culture/H1N1 |
| --- | --- | --- | --- | --- |
| Hexanoic acid | -0.437 (1.032) | 0.453 (0.742) | 0.0005 | Down |
| Lactic acid | -0.419 (0.895) | 0.434 (0.928) | 0.0008 | Down |
| RI 1947.09 | 0.415 (0.842) | -0.430 (0.980) | 0.0009 | Up |
| RI 1977.97 | 0.339 (0.786) | -0.352 (1.087) | 0.0079 | Up |
| RI 1265.13_RZ | -0.591 (0.575) | 0.613 (0.983) | < 0.0001 | Down |
| Myoinositol | 0.622 (0.735) | -0.644 (0.818) | < 0.0001 | Up |
| Serine | 0.509 (0.803) | -0.527 (0.917) | < 0.0001 | Up |
| RI 999.86 | -0.827 (0.661) | 0.857 (0.351) | < 0.0001 (W)* | Down |
| RI 990.06 | -0.541 (0.898) | 0.560 (0.773) | < 0.0001 (W) | Down |
| RI 989.84 | -0.426 (0.912) | 0.441 (0.903) | < 0.0001 (W) | Down |
| RI 989.28 | -0.641 (0.954) | 0.664 (0.480) | < 0.0001 (W) | Down |
| RI 3166.58 | 0.368 (0.978) | -0.381 (0.887) | < 0.0001 (W) | Up |
| RI 2759.58 | 0.719 (0.181) | -0.744 (0.954) | < 0.0001 (W) | Up |
| RI 2392.53 | 0.665 (0.134) | -0.689 (1.043) | < 0.0001 (W) | Up |
| RI 2117.79 | -0.521 (1.179) | 0.540 (0.195) | < 0.0001 (W) | Down |
| RI 1710.16 | -0.501 (0.858) | 0.519 (0.872) | < 0.0001 (W) | Down |
| RI 1598.51 | -0.665 (1.023) | 0.689 (0.147) | < 0.0001 (W) | Down |
| RI 1508.23 | -0.665 (0.880) | 0.689 (0.551) | < 0.0001 (W) | Down |
| RI 1321.07 | -0.712 (0.496) | 0.737 (0.843) | < 0.0001 (W) | Down |
| RI 1278.81 | -0.347 (1.314) | 0.359 (0.146) | < 0.0001 (W) | Down |
| RI 1225.56 | -0.419 (1.024) | 0.434 (0.776) | < 0.0001 (W) | Down |
| RI 1163.40 | 0.388 (1.231) | -0.402 (0.416) | < 0.0001 (W) | Up |
| RI 1089.47 | -0.283 (0.695) | 0.293 (1.183) | < 0.0001 (W) | Down |
| RI 1033.30 | 0.703 (0.619) | -0.728 (0.772) | < 0.0001 (W) | Up |
| RI 1032.37 | -0.663 (1.033) | 0.686 (0.090) | < 0.0001 (W) | Down |
| RI 1011.40 | -0.733 (0.559) | 0.759 (0.759) | < 0.0001 (W) | Down |
| RI 1008.99 | -0.853 (0.322) | 0.883 (0.613) | < 0.0001 (W) | Down |
| RI 1005.69 | 0.659 (0.317) | -0.682 (1.011) | < 0.0001 (W) | Up |
| Phenylalanine | 0.646 (0.725) | -0.669 (0.786) | < 0.0001 (W) | Up |
| Urea | 0.467 (0.622) | -0.484 (1.093) | < 0.0001 (W) | Up |
| Alanine | -0.568 (0.493) | 0.588 (1.057) | < 0.0001 (W) | Down |
| Lactic acid | -0.750 (0.620) | 0.777 (0.666) | < 0.0001 (W) | Down |
| Pyruvic acid | -0.892 (0.479) | 0.924 (0.307) | < 0.0001 (W) | Down |
| Arabinonic acid | 0.777 (0.564) | -0.805 (0.651) | < 0.0001 (W) | Up |
| Uric acid | 0.781 (0.261) | -0.809 (0.818) | < 0.0001 (W) | Up |
| Gulonic acid | -0.645 (0.750) | 0.668 (0.761) | < 0.0001 (W) | Down |
| Pentadecane, n- | -0.639 (0.857) | 0.661 (0.648) | < 0.0001 (W) | Down |
| Octadecanoic acid | 0.374 (0.221) | -0.388 (1.310) | < 0.0001 (W) | Up |
| Hexadecanoic acid | 0.457 (0.259) | -0.473 (1.244) | < 0.0001 (W) | Up |
| ALK | -0.707 (0.966) | 0.732 (0.114) | < 0.0001 (W) | Down |
| Benzoic acid, | -0.634 (0.921) | 0.657 (0.562) | < 0.0001 (W) | Down |
| Butanoic acid, 2-amino | -0.473 (1.108) | 0.489 (0.558) | < 0.0001 (W) | Down |
| Threonic acid | 0.271 (1.227) | -0.281 (0.593) | < 0.0001 (W) | Up |
| Citric acid | 0.565 (0.434) | -0.585 (1.086) | < 0.0001 (W) | Up |
| Galactose 1MEOX 5TMS BP | 0.479 (0.806) | -0.496 (0.948) | < 0.0001 (W) | Up |
| Galactose 1MEOX 5TMS MP | 0.398 (0.696) | -0.412 (1.107) | < 0.0001 (W) | Up |
| Tyrosine | 0.760 (0.410) | -0.787 (0.799) | < 0.0001 (W) | Up |
| Aspartic acid | 0.435 (0.962) | -0.450 (0.838) | < 0.0001 (W) | Up |
| Threonine | 0.238 (0.921) | -0.247 (1.034) | < 0.0001 (W) | Up |
| Lysine | 0.619 (0.752) | -0.641 (0.807) | < 0.0001 (W) | Up |
| Asparagine | 0.756 (0.250) | -0.782 (0.872) | < 0.0001 (W) | Up |
| Quinic acid | -0.134 (0.983) | 0.139 (1.016) | < 0.0001 (W) | Down |
| ALK_NA | 0.759 (0.086) | -0.786 (0.898) | < 0.0001 (W) | Up |
| ALK_NA | 0.448 (0.684) | -0.464 (1.073) | < 0.0001 (W) | Up |
| ALK_NA | 0.897 (0.370) | -0.929 (0.416) | < 0.0001 (W) | Up |
| ALK_NA | 0.679 (0.059) | -0.703 (1.031) | < 0.0001 (W) | Up |
| ALK_NA | -0.534 (0.824) | 0.553 (0.864) | < 0.0001 (W) | Down |
| ALK_NA | 0.591 (0.848) | -0.612 (0.752) | < 0.0001 (W) | Up |
| ALK_NA | 0.831 (0.270) | -0.861 (0.700) | < 0.0001 (W) | Up |
| ALK_NA | 0.556 (0.370) | -0.576 (1.121) | < 0.0001 (W) | Up |
| ALK_NA | -0.503 (1.044) | 0.521 (0.625) | < 0.0001 (W) | Down |
| ALK_NA | 0.223 (1.044) | -0.231 (0.914) | < 0.0001 (W) | Up |
| ALK_NA | 0.366 (1.056) | -0.379 (0.790) | < 0.0001 (W) | Up |
| ALK_NA | 0.766 (0.747) | -0.793 (0.461) | < 0.0001 (W) | Up |
| ALK_NA | -0.590 (0.920) | 0.611 (0.660) | < 0.0001 (W) | Down |
| Octadecadienoic acid, | 0.416 (1.008) | -0.430 (0.802) | 0.0001 (W) | Up |
| RI 1560.42 | 0.443 (0.689) | -0.459 (1.073) | 0.0002 (W) | Up |
| Sulfuric acid | -0.461 (1.215) | 0.477 (0.281) | 0.0002 (W) | Down |
| Glyceric acid | -0.457 (0.910) | 0.474 (0.870) | 0.0002 (W) | Down |
| Glycine | 0.442 (0.834) | -0.458 (0.961) | 0.0002 (W) | Up |
| ALK_NA | 0.440 (0.922) | -0.456 (0.877) | 0.0003 (W) | Up |
| Siloxane | -0.436 (1.003) | 0.452 (0.784) | 0.0003 (W) | Down |
| Alanine, beta- 1TMS | -0.381 (0.966) | 0.395 (0.888) | 0.0003 (W) | Down |
| Valine 2TMS | 0.215 (1.228) | -0.222 (0.639) | 0.0004 (W) | Up |
| RI 1133.36 | 0.200 (1.300) | -0.207 (0.485) | 0.0005 (W) | Up |
| Glutamic acid | 0.273 (0.891) | -0.283 (1.043) | 0.0005 (W) | Up |
| RI 1198.12 | -0.283 (0.770) | 0.294 (1.133) | 0.0007 (W) | Down |
| Sucrose | 0.410 (0.257) | -0.425 (1.280) | 0.0013 (W) | Up |
| Pyroglutamic acid | 0.257 (0.713) | -0.266 (1.184) | 0.0013 (W) | Up |
| Inositol like | 0.390 (0.406) | -0.404 (1.253) | 0.0022 (W) | Up |
| ALK_NA | 0.179 (1.375) | -0.185 (0.207) | 0.0033 (W) | Up |
| RI 1151.93 | 0.371 (0.991) | -0.385 (0.869) | 0.0034 (W) | Up |
| RI 3230.62 | 0.342 (1.109) | -0.354 (0.736) | 0.0040 (W) | Up |
| Homoserine 4TMS | 0.164 (0.964) | -0.170 (1.025) | 0.0043 (W) | Up |
| Homoserine | 0.161 (0.964) | -0.167 (1.026) | 0.0050 (W) | Up |
| Isoleucine | 0.324 (1.104) | -0.336 (0.762) | 0.0056 (W) | Up |
| Glucuronic acid | 0.340 (0.559) | -0.352 (1.223) | 0.0062 (W) | Up |
| RI 2678.38 | 0.023 (0.428) | -0.024 (1.372) | 0.0068 (W) | Up |
| Ethanolamine | 0.402 (0.233) | -0.416 (1.290) | 0.0068 (W) | Up |
| Psicose | 0.132 (1.249) | -0.137 (0.647) | 0.0083 (W) | Up |
| Urea | 0.383 (1.108) | -0.397 (0.693) | 0.0112 (W) | Up |
| Hexadecenoic acid | -0.305 (0.831) | 0.316 (1.074) | 0.0115 (W) | Down |
| Pyruvic acid | 0.370 (0.656) | -0.383 (1.152) | 0.0172 (W) | Up |
| Fructose | 0.353 (0.847) | -0.365 (1.029) | 0.0241 (W) | Up |
| RI 1655.22 | 0.253 (0.618) | -0.262 (1.241) | 0.0264 (W) | Up |
| Pyridine, 2-hydroxy | -0.305 (1.226) | 0.316 (0.558) | 0.0310 (W) | Down |
| Allose | -0.151 (1.016) | 0.156 (0.977) | 0.0365 (W) | Down |
| RI 818.75 | 0.314 (0.736) | -0.326 (1.138) | 0.0395 (W) | Up |

**Tables S3 and S4**. The detailed results from the univariate t-test analysis to determine significant differences of plasma metabolites/features between H1N1 and ventilated ICU controls using MetaboAnalyst software. **Table S3:** NMR data. **Table S4:** GC-MS data.

**Table S3**

| Name | Mean (SD) of H1N1 | Mean (SD) of ICU control | p-value | Change in H1N1/ICU controls |
| --- | --- | --- | --- | --- |
| O-Phosphocholine | 0.345 (1.199) | -0.431 (0.368) | 0.0003 | Up |
| Fumarate | 0.363 (0.880) | -0.454 (0.966) | 0.0004 | Up |
| Proline | 0.358 (0.963) | -0.448 (0.866) | 0.0004 | Up |
| Taurine | -0.339 (1.015) | 0.424 (0.813) | 0.0009 | Down |
| Choline | 0.328 (0.882) | -0.410 (0.999) | 0.0014 | Up |
| 2-Hydroxyisovalerate | 0.293 (1.110) | -0.366 (0.700) | 0.0031 | Up |
| 2-Oxoglutarate | -0.293 (0.908) | 0.367 (1.002) | 0.0046 | Down |
| Glycine | -0.283 (1.024) | 0.354 (0.860) | 0.0064 | Down |
| Alanine | -0.263 (0.944) | 0.329 (0.983) | 0.0115 | Down |
| Tyrosine | -0.240 (1.180) | 0.300 (0.608) | 0.0149 | Down |
| Succinate | 0.236 (1.087) | -0.294 (0.801) | 0.0244 | Up |
| Pyruvate | 0.219 (0.965) | -0.274 (0.989) | 0.0364 | Up |
| 3-Hydroxyisovalerate | 0.394 (0.985) | -0.493 (0.787) | < 0.0001 | Up |
| Aspartate | 0.525 (0.929) | -0.656 (0.637) | < 0.0001 | Up |
| Formate | 0.405 (1.022) | -0.506 (0.708) | < 0.0001 | Up |
| Isopropanol | -0.512 (0.822) | 0.640 (0.824) | < 0.0001 | Down |
| Phenylalanine | 0.543 (0.912) | -0.679 (0.622) | < 0.0001 | Up |
| Citrate | -0.399 (0.934) | 0.499 (0.854) | < 0.0001 (W) | Down |
| Dimethylamine | 0.536 (0.699) | -0.671 (0.916) | < 0.0001 (W) | Up |
| Beta Alanine | 0.565 (0.957) | -0.706 (0.460) | < 0.0001 (W) | Up |
| Adipate | 0.322 (1.196) | -0.403 (0.434) | 0.0003 (W) | Up |
| Ornithine | 0.343 (0.873) | -0.429 (0.994) | 0.0006 (W) | Up |
| 3-Methyl-2-oxovalerate | 0.274 (0.894) | -0.343 (1.033) | 0.0014 (W) | Up |
| Glutamine | -0.302 (1.152) | 0.377 (0.597) | 0.0035 (W) | Down |
| Carnitine | 0.203 (1.131) | -0.254 (0.749) | 0.0090 (W) | Up |
| Isobutyrate | 0.223 (1.056) | -0.279 (0.863) | 0.0114 (W) | Up |
| Asparagine | -0.189 (1.108) | 0.237 (0.802) | 0.0131 (W) | Down |

**Table S4**

| Name* | Mean (SD) of H1N1 | Mean (SD) of ICU | p-value | Change in H1N1/ICU |
| --- | --- | --- | --- | --- |
| Octadecadienoic acid, | -0.257 (1.227) | 0.348 (0.351) | 0.0038 | Down |
| Isoleucine 2TMS | 0.709 (0.609) | -0.960 (0.485) | < 0.0001 | Up |
| Fructose | 0.570 (0.968) | -0.772 (0.222) | < 0.0001 | Up |
| Octadecenoic acid | -0.549 (0.894) | 0.744 (0.559) | < 0.0001 | Down |
| Lactic acid | 0.801 (0.295) | -1.086 (0.404) | < 0.0001 | Up |
| RI 997.09 | 0.701 (0.142) | -0.950 (0.867) | < 0.0001 (W) | Up |
| RI 995.60 | 0.758 (0.441) | -1.027 (0.488) | < 0.0001 (W) | Up |
| RI 991.09 | 0.552 (0.457) | -0.748 (1.054) | < 0.0001 (W) | Up |
| RI 964.76 | 0.812 (0.315) | -1.100 (0.304) | < 0.0001 (W) | Up |
| RI 960.51 | -0.597 (0.944) | 0.809 (0.082) | < 0.0001 (W) | Down |
| RI 2390.97 | -0.642 (0.868) | 0.870 (0.097) | < 0.0001 (W) | Down |
| RI 2341.35 | 0.138 (1.265) | -0.186 (0.386) | < 0.0001 (W) | Up |
| RI 2162.25 | -0.150 (1.052) | 0.203 (0.902) | < 0.0001 (W) | Down |
| RI 2124.20 | -0.469 (0.976) | 0.635 (0.610) | < 0.0001 (W) | Down |
| RI 1955.44 | -0.516 (0.885) | 0.699 (0.672) | < 0.0001 (W) | Down |
| RI 1751.77 | -0.071 (0.312) | 0.096 (1.500) | < 0.0001 (W) | Down |
| RI 1508.23 | 0.570 (0.196) | -0.772 (1.129) | < 0.0001 (W) | Up |
| RI 1507.96 | 0.538 (0.182) | -0.729 (1.183) | < 0.0001 (W) | Up |
| RI 1278.87 | 0.458 (0.853) | -0.620 (0.845) | < 0.0001 (W) | Up |
| RI 1056.65 | 0.792 (0.413) | -1.073 (0.314) | < 0.0001 (W) | Up |
| RI 1053.41 | -0.297 (1.107) | 0.403 (0.659) | < 0.0001 (W) | Down |
| RI 1050.89 | 0.708 (0.182) | -0.959 (0.838) | < 0.0001 (W) | Up |
| RI 1045.85 | 0.789 (0.190) | -1.070 (0.544) | < 0.0001 (W) | Up |
| RI 1030.80 | 0.500 (0.967) | -0.677 (0.545) | < 0.0001 (W) | Up |
| RI 1009.17 | 0.374 (1.087) | -0.507 (0.569) | < 0.0001 (W) | Up |
| RI 1007.58 | 0.826 (0.194) | -1.119 (0.319) | < 0.0001 (W) | Up |
| Phosphoric acid | 0.075 (1.305) | -0.101 (0.230) | < 0.0001 (W) | Up |
| Benzoic acid | 0.070 (1.295) | -0.094 (0.302) | < 0.0001 (W) | Up |
| Urea | 0.468 (0.885) | -0.635 (0.778) | < 0.0001 (W) | Up |
| Butanoic acid, 2-hydroxy | 0.611 (0.873) | -0.828 (0.358) | < 0.0001 (W) | Up |
| Octadecan-1-ol, | -0.515 (0.895) | 0.698 (0.655) | < 0.0001 (W) | Down |
| Fructose | 0.491 (0.639) | -0.666 (1.022) | < 0.0001 (W) | Up |
| Octadecenoic acid | -0.452 (0.890) | 0.612 (0.803) | < 0.0001 (W) | Down |
| Octadecanoic acid | -0.437 (1.133) | 0.593 (0.127) | < 0.0001 (W) | Down |
| Heptadecanoic acid | -0.293 (1.243) | 0.397 (0.067) | < 0.0001 (W) | Down |
| Hexadecanoic acid | -0.567 (0.977) | 0.768 (0.193) | < 0.0001 (W) | Down |
| Butanoic acid, 3-hydroxy | 0.471 (1.050) | -0.639 (0.401) | < 0.0001 (W) | Up |
| Benzoic acid | 0.427 (0.894) | -0.579 (0.841) | < 0.0001 (W) | Up |
| Glycerol-3-phosphate | -0.098 (0.476) | 0.132 (1.435) | < 0.0001 (W) | Down |
| Galactopyranoside, 1-O-methyl-, beta | 0.735 (0.181) | -0.996 (0.756) | < 0.0001 (W) | Up |
| Phosphoric acid | -0.614 (0.910) | 0.831 (0.166) | < 0.0001 (W) | Down |
| Glyceric acid | 0.615 (0.355) | -0.833 (0.991) | < 0.0001 (W) | Up |
| Glycerol | 0.495 (0.850) | -0.671 (0.777) | < 0.0001 (W) | Up |
| Galactose | 0.822 (0.307) | -1.113 (0.213) | < 0.0001 (W) | Up |
| Galactose | 0.510 (1.007) | -0.691 (0.397) | < 0.0001 (W) | Up |
| Glucose | 0.685 (0.764) | -0.928 (0.239) | < 0.0001 (W) | Up |
| Pyroglutamic acid | 0.736 (0.372) | -0.997 (0.651) | < 0.0001 (W) | Up |
| Glycine | 0.666 (0.219) | -0.902 (0.934) | < 0.0001 (W) | Up |
| Alanine, beta | 0.584 (0.267) | -0.791 (1.085) | < 0.0001 (W) | Up |
| Quinic acid | -0.147 (0.512) | 0.199 (1.404) | < 0.0001 (W) | Down |
| Pentadecane, n- | -0.300 (1.173) | 0.406 (0.472) | 0.0001 (W) | Down |
| Octadecanoic acid | 0.425 (1.089) | -0.576 (0.429) | 0.0002 (W) | Up |
| RI 1530.64 | 0.252 (0.945) | -0.341 (0.985) | 0.0008 (W) | Up |
| RI 987.26 | 0.244 (1.019) | -0.331 (0.885) | 0.0010 (W) | Up |
| Heptadecane, n- | -0.254 (1.178) | 0.344 (0.539) | 0.0011 (W) | Down |
| Mannose | 0.390 (1.160) | -0.529 (0.242) | 0.0172 (W) | Up |
| RI 1793.17 | -0.020 (0.915) | 0.028 (1.120) | 0.0383 (W) | Down |

RI* show the retention index for unknown features in GC-MS analysis

**Tables S5 and S6**. The detailed results from the univariate t-test analysis to determine significant differences of plasma metabolites/features between H1N1 non-survivor and survivors using MetaboAnalyst software. **Table S5:** NMR data. **Table S6:** GC-MS data.

**Table S5**

| Name | Mean (SD) of Nonsurvivors | Mean (SD) of Survivors | p-value | Change in Nonsurvivors/Survivors |
| --- | --- | --- | --- | --- |
| 2-Oxoglutarate | -0.797 (0.814) | 0.399 (0.848) | 0.0061 | Down |
| Acetoacetate | 0.599 (0.948) | -0.299 (0.913) | 0.0495 | Up |
| 2-Hydroxyisovalerate | -0.583 (0.671) | 0.292 (1.027) | 0.0563 | Down |
| Arginine | 0.564 (0.786) | -0.282 (0.998) | 0.0656 | Up |
| Isopropanol | -0.533 (1.094) | 0.266 (0.870) | 0.084 | Down |
| 2-Hydroxybutyrate | 0.519 (0.932) | -0.260 (0.959) | 0.0929 | Up |
| Isobutyrate | -0.411 (0.852) | 0.206 (1.033) | 0.0793 (W) | Down |

**Table S6**

| Name* | Mean (SD) of Nonsurvivors | Mean (SD) of Survivors | p-value | Change in Nonsurvivors/Survivors |
| --- | --- | --- | --- | --- |
| RI 1144.7 | -0.219 (1.141) | 0.438 (0.419) | 0.0718 | Down |
| Isoleucine | 0.262 (0.789) | -0.525 (1.226) | 0.0889 | Up |
| RI 1262.47 | -0.262 (0.912) | 0.524 (1.024) | 0.0895 | Down |
| RI 1128.87 | 0.506 (0.844) | -1.012 (0.016) | 0.0016 (W) | Up |
| RI 1337.52 | 0.372 (1.044) | -0.744 (0.014) | 0.0125 (W) | Up |
| RI 1885.03 | 0.408 (1.000) | -0.817 (0.012) | 0.0250 (W) | Up |
| Valine | 0.313 (0.820) | -0.625 (1.092) | 0.0309 (W) | Up |
| RI 1671.4 | -0.291 (1.111) | 0.582 (0.255) | 0.0461 (W) | Down |
| Hydroxylamine | -0.235 (1.121) | 0.470 (0.472) | 0.0461 (W) | Down |
| Pyruvic acid, 4-hydroxyphenyl- | 0.277 (0.952) | -0.553 (0.912) | 0.0556 (W) | Up |
| Hexadecane | -0.248 (0.923) | 0.495 (1.028) | 0.0556 (W) | Down |
| RI 1122.27 | 0.190 (1.074) | -0.381 (0.763) | 0.0667 (W) | Up |
| RI 1818.38 | 0.272 (1.140) | -0.545 (0.014) | 0.0793 (W) | Up |
| Galactose | 0.248 (0.883) | -0.495 (1.103) | 0.0793 (W) | Up |
| RI 1410.07 | 0.180 (1.058) | -0.361 (0.824) | 0.0938 (W) | Up |
| RI 2014.28 | 0.265 (0.507) | -0.530 (1.512) | 0.0938 (W) | Up |

RI* show the retention index for unknown features in GC-MS analysis

**Tables S7 and S8** show the detailed results from the MetaboAnalyst pathway analysis of plasma metabolomics analysis of H1N1 patients vs. positive bacterial culture CAP patients. Since many pathways have been tested at the same time, the statistical p values from enrichment analysis are further adjusted for multiple testing. In particular, the Total is the total number of compounds in the pathway; the Hits is the actually matched number from the user uploaded data; the Raw p is the original p value calculated from the enrichment analysis; the Holm p is the p value adjusted by Holm-Bonferroni method; the FDR p is the p value adjusted using False Discovery Rate; the Impact is the pathway impact value calculated from pathway topology analysis. Most reliably affected biological pathways are bold in the tables. These pathways have an impact > 0.1.

**Table S7.** Result from Pathways Analysis of NMR data for H1N1 patients vs. positive bacterial culture CAP.

|  | Pathway names | Total | Expected | Hits | Raw p | LOG(p) | Holm adjust | FDR | Impact |
| --- | --- | --- | --- | --- | --- | --- | --- | --- | --- |
| 1 | **Lysine degradation** | 47 | **0.234** | **1** | **0.211** | **1.555** | **1** | **0.939** | **0.146** |
| 2 | **Inositol phosphate metabolism** | 39 | **0.194** | **1** | **0.178** | **1.723** | **1** | **0.939** | **0.137** |
| 3 | Lysine biosynthesis | 32 | 0.159 | 1 | 0.148 | 1.905 | 1 | 0.939 | 0.099 |
| 4 | Glycine, serine and threonine metabolism | 48 | 0.239 | 1 | 0.215 | 1.536 | 1 | 0.939 | 0.096 |
| 5 | Pentose phosphate pathway | 32 | 0.159 | 1 | 0.148 | 1.905 | 1 | 0.939 | 0.086 |
| 6 | Alanine, aspartate and glutamate metabolism | 24 | 0.119 | 2 | 0.005 | 5.129 | 0.467 | 0.236 | 0.068 |
| 7 | Citrate cycle (TCA cycle) | 20 | 0.099 | 1 | 0.095 | 2.348 | 1 | 0.939 | 0.063 |
| 8 | Aminoacyl-tRNA biosynthesis | 75 | 0.373 | 4 | 0.000 | 7.939 | 0.028 | 0.028 | 0.056 |
| 9 | Tyrosine metabolism | 76 | 0.378 | 1 | 0.320 | 1.138 | 1 | 1 | 0.047 |
| 10 | Purine metabolism | 92 | 0.458 | 1 | 0.374 | 0.982 | 1 | 1 | 0.009 |
| 11 | Ascorbate and aldarate metabolism | 45 | 0.224 | 2 | 0.020 | 3.910 | 1 | 0.400 | 0.008 |
| 12 | Nitrogen metabolism | 39 | 0.194 | 2 | 0.015 | 4.183 | 1 | 0.400 | 0.007 |
| 13 | Phenylalanine, tyrosine and tryptophan biosynthesis | 27 | 0.134 | 1 | 0.126 | 2.064 | 1 | 0.939 | 0.007 |
| 14 | Glyoxylate and dicarboxylate metabolism | 50 | 0.249 | 1 | 0.223 | 1.5 | 1 | 0.939 | 0.003 |
| 15 | Biotin metabolism | 11 | 0.054 | 1 | 0.053 | 2.926 | 1 | 0.857 | 0 |
| 16 | Cyanoamino acid metabolism | 16 | 0.079 | 1 | 0.077 | 2.562 | 1 | 0.939 | 0 |
| 17 | Thiamine metabolism | 24 | 0.119 | 1 | 0.113 | 2.175 | 1 | 0.939 | 0 |
| 181 | Valine, leucine and isoleucine biosynthesis | 27 | 0.134 | 1 | 0.126 | 2.064 | 1 | 0.939 | 0 |
| 9 | Ubiquinone and other terpenoid-quinone biosynthesis | 36 | 0.179 | 1 | 0.165 | 1.797 | 1 | 0.939 | 0 |
| 20 | Galactose metabolism | 41 | 0.204 | 1 | 0.186 | 1.678 | 1 | 0.939 | 0 |
| 21 | Phenylalanine metabolism | 45 | 0.224 | 1 | 0.203 | 1.594 | 1 | 0.939 | 0 |
| 22 | Porphyrin and chlorophyll metabolism | 104 | 0.518 | 1 | 0.412 | 0.886 | 1 | 1 | 0 |

**Table S8.** Result from Pathways Analysis of GC-MS data for H1N1 patients vs. positive bacterial culture CAP patients.

|  | Pathway names | Total | Expected | Hits | Raw p | LOG(p) | Holm adjust | FDR | Impact |
| --- | --- | --- | --- | --- | --- | --- | --- | --- | --- |
| 1 | Synthesis and degradation of ketone bodies | 6 | 0.027 | 1 | 0.0277 | 3.606 | 1 | 0.217 | 0.7 |
| 2 | beta-Alanine metabolism | 28 | 0.127 | 2 | 0.006 | 5.001 | 0.504 | 0.089 | 0.256 |
| 3 | Glycine, serine and threonine metabolism | 48 | 0.219 | 1 | 0.199 | 1.613 | 1 | 0.636 | 0.187 |
| 4 | Methane metabolism | 34 | 0.155 | 3 | 0.0003 | 7.840 | 0.030 | 0.010 | 0.146 |
| 5 | Glyoxylate and dicarboxylate metabolism | 50 | 0.228 | 1 | 0.206 | 1.577 | 1 | 0.635 | 0.143 |
| 6 | Histidine metabolism | 44 | 0.201 | 1 | 0.184 | 1.692 | 1 | 0.635 | 0.139 |
| 7 | Propanoate metabolism | 35 | 0.159 | 2 | 0.010 | 4.565 | 0.759 | 0.104 | 0.113 |
| 8 | Pyruvate metabolism | 32 | 0.146 | 2 | 0.008 | 4.739 | 0.646 | 0.099 | 0.099 |
| 9 | Pantothenate and CoA biosynthesis | 27 | 0.123 | 1 | 0.116 | 2.146 | 1 | 0.584 | 0.072 |
| 10 | Butanoate metabolism | 40 | 0.182 | 2 | 0.013 | 4.307 | 0.970 | 0.119 | 0.069 |
| 11 | Tyrosine metabolism | 76 | 0.347 | 2 | 0.045 | 3.101 | 1 | 0.327 | 0.042 |
| 12 | Cysteine and methionine metabolism | 56 | 0.255 | 1 | 0.228 | 1.476 | 1 | 0.677 | 0.038 |
| 13 | Sulfur metabolism | 18 | 0.082 | 1 | 0.0791 | 2.531 | 1 | 0.529 | 0.033 |
| 14 | Primary bile acid biosynthesis | 47 | 0.214 | 1 | 0.195 | 1.632 | 1 | 0.635 | 0.008 |
| 15 | Phenylalanine, tyrosine and tryptophan biosynthesis | 27 | 0.123 | 1 | 0.116 | 2.146 | 1 | 0.584 | 0.007 |
| 16 | Selenoamino acid metabolism | 22 | 0.100 | 1 | 0.096 | 2.340 | 1 | 0.550 | 0.003 |
| 17 | Glycolysis or Gluconeogenesis | 31 | 0.141 | 1 | 0.133 | 2.016 | 1 | 0.626 | 0.000 |
| 18 | Nitrogen metabolism | 39 | 0.178 | 4 | 0.0001 | 10.92 | 0.001 | 0.001 | 0 |
| 19 | Aminoacyl-tRNA biosynthesis | 75 | 0.342 | 4 | 0.000 | 8.321 | 0.019 | 0.009 | 0 |
| 20 | Cyanoamino acid metabolism | 16 | 0.073 | 2 | 0.002 | 6.118 | 0.169 | 0.0440 | 0 |
| 21 | Thiamine metabolism | 24 | 0.109 | 2 | 0.004 | 5.305 | 0.377 | 0.079 | 0 |
| 22 | Taurine and hypotaurine metabolism | 20 | 0.091 | 1 | 0.087 | 2.431 | 1 | 0.540 | 0 |
| 23 | Ubiquinone and other terpenoid-quinone biosynthesis | 36 | 0.164 | 1 | 0.153 | 1.877 | 1 | 0.635 | 0 |
| 24 | Glutathione metabolism | 38 | 0.173 | 1 | 0.169 | 1.827 | 1 | 0.635 | 0 |
| 25 | Valine, leucine and isoleucine degradation | 40 | 0.182 | 1 | 0.168 | 1.779 | 1 | 0.635 | 0 |
| 26 | Phenylalanine metabolism | 45 | 0.205 | 1 | 0.187 | 1.672 | 1 | 0.635 | 0 |
| 27 | Lysine degradation | 47 | 0.214 | 1 | 0.195 | 1.632 | 1 | 0.635 | 0 |
| 28 | Pyrimidine metabolism | 60 | 0.274 | 1 | 0.242 | 1.415 | 1 | 0.694 | 0 |
| 29 | Purine metabolism | 92 | 0.420 | 1 | 0.349 | 1.052 | 1 | 0.963 | 0 |
| 30 | Porphyrin and chlorophyll metabolism | 104 | 0.475 | 1 | 0.385 | 0.953 | 1 | 1 | 0 |

**Figure S11.** Summary of pathway analysis with MetaboAnalyst for the diagnosis of H1N1 patients from positive bacterial culture CAP patients. Each circle is a representative of a biological pathway and the size of the circle is enumerated based on the importance. **A:** summary of pathway analysis with MetaboAnalyst based on NMR data and **B:** GC-MS data. The number of the pathway corresponds to the number of the pathway shown in Tables S8 (NMR) and S9 (GC-MS).


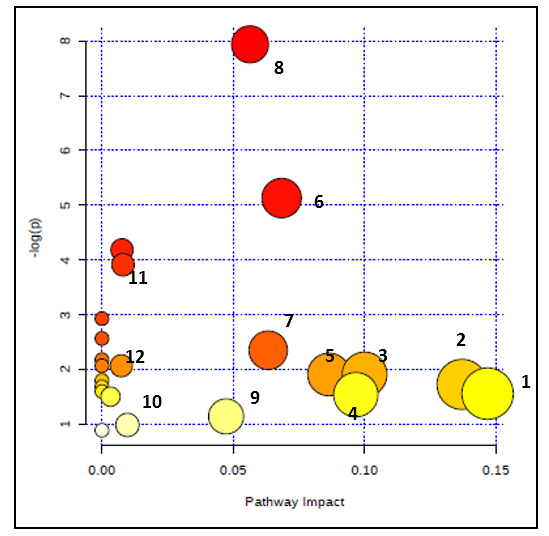


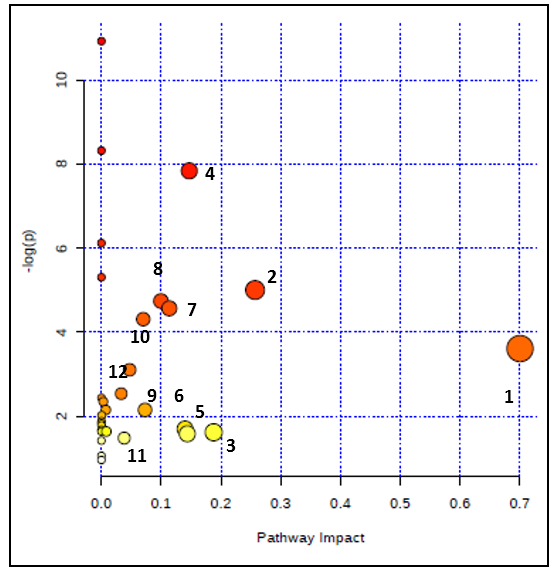


**Figure S11A Figure S11B**

**Tables S9 and S10** show the detailed results from the MetaboAnalyst pathway analysis of plasma metabolomics from H1N1 patients vs. ICU ventilated controls. Since many pathways have been tested at the same time, the statistical p values from enrichment analysis are further adjusted for multiple testing. In particular, the Total is the total number of compounds in the pathway; the Hits is the actually matched number from the user uploaded data; the Raw p is the original p value calculated from the enrichment analysis; the Holm p is the p value adjusted by Holm-Bonferroni method; the FDR p is the p value adjusted using False Discovery Rate; the Impact is the pathway impact value calculated from pathway topology analysis. Most reliably affected biological pathways are bold in the tables. These pathways have an impact > 0.1.

**Table S9.** Result from Pathways Analysis of NMR data for diagnosis of H1N1 from ICU ventilated control study

|  | Pathway names | Total | Expected | Hits | Raw p | Log (0) | Holm adjust | FDR | Impact |
| --- | --- | --- | --- | --- | --- | --- | --- | --- | --- |
| 11  1 | **Taurine and hypotaurine metabolism** | **20** | **0.17** | **1** | **0.161** | **1.82** | **1** | **0.496** | **0.33** |
| 22 | **D-Glutamine and D-glutamate**  **metabolism** | **11** | **0.10** | **2** | **0.003** | **5.57** | **0.266** | **0.027** | **0.33** |
| 33 | **Glycine, serine and threonine metabolism** | **48** | **0.42** | **3** | **0.007** | **4.87** | **0.523** | **0.047** | **0.32** |
| 44 | **Alanine, aspartate and glutamate**  **metabolism** | **24** | **0.21** | **5** | **<0.0001** | **0.137** | **<0.0001** | **<0.0001** | **0.31** |
| 55 | **beta-Alanine metabolism** | **28** | **0.24** | **2** | **0.023** | **3.73** | **1** | **0.120** | **0.26** |
| 66 | **Citrate cycle (TCA cycle)** | **20** | **0.17** | **4** | **<0.0001** | **1.09** | **0.0144** | **<0.0001** | **0.18** |
| 77 | **Methane metabolism** | **34** | **0.30** | **4** | **<0.0001** | **8.69** | **0.0126** | **0.002** | **0.16** |
| 88 | **Glyoxylate and dicarboxylate metabolism** | **50** | **0.44** | **4** | **0.0007** | **7.18** | **0.0548** | **0.006** | **0.15** |
| 99 | **Phenylalanine metabolism** | **45** | **0.39** | **4** | **0.0005** | **7.59** | **0.0370** | **0.005** | **0.12** |
| 110 | **Aminoacyl-tRNA biosynthesis** | **75** | **0.65** | **8** | **<0.0001** | **0.162** | **<0.0001** | **<0.0001** | **0.11** |
| 111 | **Arginine and proline metabolism** | **77** | **0.67** | **3** | **0.027** | **3.59** | **1** | **0.123** | **0.10** |
| 112 | Propanoate metabolism | 35 | 0.31 | 4 | 0.0001 | 8.58 | 0.013 | 0.002 | 0.09 |
| 113 | Pantothenate and CoA biosynthesis | 27 | 0.24 | 3 | 0.001 | 6.53 | 0.104 | 0.011 | 0.07 |
| 114 | Tyrosine metabolism | 76 | 0.66 | 3 | 0.026 | 3.62 | 1 | 0.123 | 0.05 |
| 115 | Glycerophospholipid metabolism | 39 | 0.34 | 1 | 0.291 | 1.23 | 1 | 0.722 | 0.05 |
| 116 | Vitamin B6 metabolism | 32 | 0.28 | 2 | 0.030 | 3.48 | 1 | 0.123 | 0.03 |
| 117 | Primary bile acid biosynthesis | 47 | 0.41 | 2 | 0.061 | 2.78 | 1 | 0.215 | 0.02 |
| 118 | Cysteine and methionine metabolism | 56 | 049 | 2 | 0.084 | 2.47 | 1 | 0.281 | 0.01 |
| 119 | Valine, leucine and isoleucine biosynthesis | 27 | 0.24 | 1 | 0.212 | 1.55 | 1 | 0.605 | 0.01 |
| 220 | Phenylalanine, tyrosine and tryptophan  biosynthesis | 27 | 0.24 | 2 | 0.0223 | 3.80 | 1 | 0.119 | 0.01 |
| 221 | Nitrogen metabolism | 39 | 0.34 | 7 | <0.0001 | 0.179 | <0.0001 | 0.132 | 0.01 |

**Table S10.** Result from Pathways Analysis of GC-MS data for diagnosis of H1N1 from ICU ventilated control study.

|  | Pathway names | Total | Expected | Hits | Raw p | Log (0) | Holm adjust | FDR | Impact |
| --- | --- | --- | --- | --- | --- | --- | --- | --- | --- |
| 11  1 | **beta-Alanine metabolism** | 28 | 0.19 | 1 | 0.180 | 1.709 | 1 | 0.904 | 0.256 |
| 22 | **Glycine, serine and threonine metabolism** | **48** | **0.33** | **2** | **0.043** | **3.128** | **1** | **0.377** | **0.188** |
| 33 | **Pyruvate metabolism** | **32** | **0.22** | **1** | **0.204** | **1.589** | **1** | **0.907** | **0.138** |
| 44 | Propanoate metabolism | 35 | 0.247 | 2 | 0.024 | 3.714 | 1 | 0.374 | 0.085 |
| 55 | Pantothenate and CoA biosynthesis | 27 | 0.190 | 1 | 0.175 | 1.742 | 1 | 0.904 | 0.072 |
| 66 | Glyoxylate and dicarboxylate metabolism | 50 | 0.353 | 1 | 0.300 | 1.200 | 1 | 0.926 | 0.032 |
| 77 | Arginine and proline metabolism | 77 | 0.543 | 1 | 0.425 | 0.854 | 1 | 1 | 0.030 |
| 88 | Fatty acid metabolism | 50 | 0.352 | 1 | 0.300 | 1.200 | 1 | 0.926 | 0.029 |
| 99 | Fructose and mannose metabolism | 48 | 0.339 | 1 | 0.290 | 1.235 | 1 | 0.926 | 0.029 |
| 110 | Pentose phosphate pathway | 32 | 0.226 | 2 | 0.020 | 3.884 | 1 | 0.374 | 0.021 |
| 111 | Glycerolipid metabolism | 32 | 0.226 | 1 | 0.204 | 1.589 | 1 | 0.907 | 0.020 |
| 112 | Starch and sucrose metabolism | 50 | 0.353 | 2 | 0.047 | 3.054 | 1 | 0.377 | 0.017 |
| 113 | Primary bile acid biosynthesis | 47 | 0.331 | 1 | 0.285 | 1.253 | 1 | 0.926 | 0.008 |
| 114 | Purine metabolis | 92 | 0.649 | 2 | 0.135 | 1.99 | 1 | 0.904 | 0.007 |
| 115 | Galactose metabolism | 41 | 0.289 | 2 | 0.032 | 3.418 | 1 | 0.374 | 0.002 |
| 116 | Glutathione metabolism | 38 | 0.268 | 2 | 0.028 | 3.5601 | 1 | 0.374 | 0.001 |

**Figure S12.** Summary of pathway analysis with MetaboAnalyst for the diagnosis of H1N1from ICU ventilated control. Each circle is a representative of a biological pathway and the size of the circle is enumerated based on the importance. A: summary of pathway analysis with MetaboAnalyst based on NMR data and B: GC-MS data. The number of the pathway corresponds to the number of the pathway shown in Tables S10 (NMR) and S11(GC-MS).


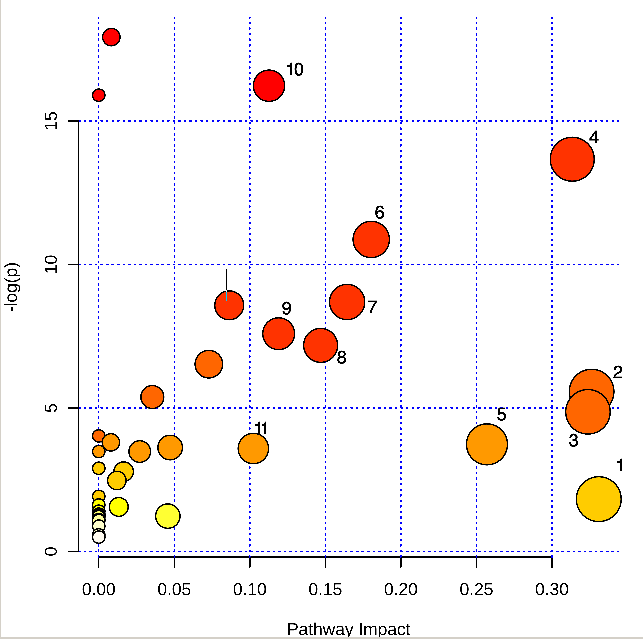

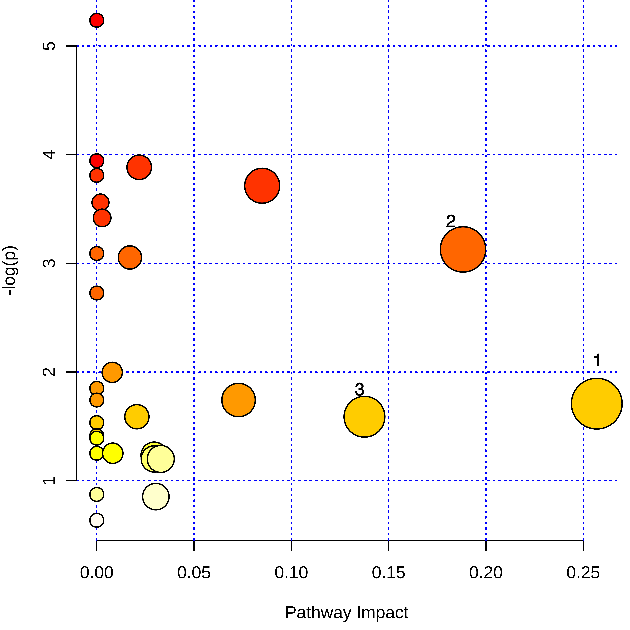


Figure S 12A Figure S 12B

**Tables S11 and S12** show the detailed results from the MetaboAnalyst pathway analysis of plasma from H1N1 survivors vs. non-survivors. Since many pathways have been tested at the same time, the statistical p values from enrichment analysis are further adjusted for multiple testing. In particular, the Total is the total number of compounds in the pathway; the Hits is the actually matched number from the user uploaded data; the Raw p is the original p value calculated from the enrichment analysis; the Holm p is the p value adjusted by Holm-Bonferroni method; the FDR p is the p value adjusted using False Discovery Rate; the Impact is the pathway impact value calculated from pathway topology analysis. Most reliably affected biological pathways are bold in the tables. These pathways have an impact > 0.1.

**Table S11.** Result from Pathways Analysis of NMR data for survivors vs. non-survivors of H1N1.

| \|  \| Pathway names \| Total \| Expected \| Hits \| Raw p \| Log (0) \| Holm adjust \| FDR \| Impact \| \| --- \| --- \| --- \| --- \| --- \| --- \| --- \| --- \| --- \| --- \| \| 1 \| **Synthesis and degradation of ketone bodies** \| **6** \| **0.044** \| **2** \| **0.0007** \| **7.158** \| **0.060** \| **0.020** \| **0.7** \| \| 2 \| **D-Arginine and D-Ornithine metabolism** \| **8** \| **0.059** \| **2** \| **0.001** \| **6.542** \| **0.110** \| **0.028** \| **0.5** \| \| 3 \| **Pyruvate metabolism** \| **32** \| **0.239** \| **2** \| **0.022** \| **3.774** \| **1** \| **0.143** \| **0.23** \| \| 4 \| **Arginine and Proline metabolism** \| **77** \| **0.575** \| **4** \| **0.002** \| **6.162** \| **0.158** \| **0.028** \| **0.23** \| \| 5 \| **Phenylalanine metabolism** \| **45** \| **0.336** \| **3** \| **0.004** \| **5.497** \| **0.303** \| **0.046** \| **0.11** \| \| 6 \| **Citrate cycle (TCA cycle)** \| **20** \| **0.149** \| **2** \| **0.009** \| **4.681** \| **0.676** \| **0.092** \| **0.10** \| \| 7 \| Butanoate metabolism \| 40 \| 0.299 \| 4 \| 0.0001 \| 8.683 \| 0.013 \| 0.006 \| 0.06 \| \| 8 \| Tyrosine metabolism \| 76 \| 0.568 \| 3 \| 0.017 \| 4.042 \| 1 \| 0.127 \| 0.04 \| \| 9 \| Cysteine and Methionine metabolism \| 56 \| 0.418 \| 1 \| 0.346 \| 1.060 \| 1 \| 0.893 \| 0.03 \| \| 10 \| Sulfur metabolism \| 18 \| 0.134 \| 1 \| 0.126 \| 2.065 \| 1 \| 0.634 \| 0.03 \| \| 11 \| Fructose and Mannose metabolism \| 48 \| 0.358 \| 1 \| 0.305 \| 1.187 \| 1 \| 0.841 \| 0.02 \| \| 12 \| Propanoate metabolism \| 35 \| 0.261 \| 3 \| 0.001 \| 6.224 \| 0.150 \| 0.028 \| 0.02 \| \| 13 \| Vitamin B6 metabolism \| 32 \| 0.239 \| 1 \| 0.214 \| 1.538 \| 1 \| 0.818 \| 0.01 \| \| 14 \| Phenylalanine, Tyrosine and Tryptophan biosynthesis \| 27 \| 0.201 \| 2 \| 0.016 \| 4.098 \| 1 \| 0.127 \| 0.008 \| \| 15 \| Selenoamino acid metabolism \| 22 \| 0.164 \| 1 \| 0.152 \| 1.878 \| 1 \| 0.679 \| 0.003 \| \| 16 \| Alanine, Asparate and Glutamate metabolism \| 24 \| 0.179 \| 2 \| 0.011 \| 4.325 \| 0.952 \| 0.117 \| 0.002 \| \| 17 \| Methane metabolism \| 34 \| 0.254 \| 1 \| 0.226 \| 1.484 \| 1 \| 0.824 \| 0.0005 \| \| 18 \| Glycolysis or Gluconeogenesis \| 31 \| 0.231 \| 2 \| 0.021 \| 3.834 \| 1 \| 0.141 \| 0.0004 \| |  |  |  |  |  |  |  |  |
| --- | --- | --- | --- | --- | --- | --- | --- | --- | --- | --- | --- | --- | --- | --- | --- | --- | --- | --- | --- | --- | --- | --- | --- | --- | --- | --- | --- | --- | --- | --- | --- | --- | --- | --- | --- | --- | --- | --- | --- | --- | --- | --- | --- | --- | --- | --- | --- | --- | --- | --- | --- | --- | --- | --- | --- | --- | --- | --- | --- | --- | --- | --- | --- | --- | --- | --- | --- | --- | --- | --- | --- | --- | --- | --- | --- | --- | --- | --- | --- | --- | --- | --- | --- | --- | --- | --- | --- | --- | --- | --- | --- | --- | --- | --- | --- | --- | --- | --- | --- | --- | --- | --- | --- | --- | --- | --- | --- | --- | --- | --- | --- | --- | --- | --- | --- | --- | --- | --- | --- | --- | --- | --- | --- | --- | --- | --- | --- | --- | --- | --- | --- | --- | --- | --- | --- | --- | --- | --- | --- | --- | --- | --- | --- | --- | --- | --- | --- | --- | --- | --- | --- | --- | --- | --- | --- | --- | --- | --- | --- | --- | --- | --- | --- | --- | --- | --- | --- | --- | --- | --- | --- | --- | --- | --- | --- | --- | --- | --- | --- | --- | --- | --- | --- | --- | --- | --- | --- | --- | --- | --- | --- | --- | --- | --- | --- | --- | --- | --- |

**Table S12**: Result from Pathway Analysis of GC-MS data for survivors vs. non-survivors of H1N1.

|  | Pathway names | Total | Expected | Hits | Raw p | -Log (0) | Holm adjust | FDR | Impact |
| --- | --- | --- | --- | --- | --- | --- | --- | --- | --- |
| 11 | **Beta-Alanine metabolism** | **28** | **0.244** | **1** | **0.218** | **1.520** | **1** | **0.728** | **0.256** |
| 22 | **Galactose metabolism** | **41** | **0.357** | **4** | **0.0003** | **7.951** | **0.028** | **0.028** | **0.226** |
| 33 | **Glycerolipid metabolism** | **32** | **0.279** | **2** | **0.017** | **3.482** | **1** | **0.143** | **0.237** |
| 44 | **Alanine, Asparate and Glutamate metabolism** | **24** | **0.209** | **2** | **0.017** | **4.026** | **1** | **0.178** | **0.207** |
| 55 | **Pyruvate metabolism** | **32** | **0.279** | **1** | **0.245** | **1.402** | **1** | **0.748** | **0.182** |
| 56 | Glycolysis or Gluconeogenesis | 31 | 0.270 | 3 | 0.002 | 6.119 | 0.169 | 0.038 | 0.095 |
| 67 | Citrate cycle (TCA cycle) | 20 | 0.174 | 1 | 0.161 | 1.824 | 1 | 0.586 | 0.090 |
| 78 | Butanoate metabolism | 40 | 0.348 | 2 | 0.046 | 3.071 | 1 | 0.285 | 0.085 |
| 89 | Propanoate metabolism | 35 | 0.305 | 2 | 0.036 | 3.316 | 1 | 0.285 | 0.085 |
| 910 | Panththenate and CoA biosynthesis | 27 | 0.236 | 3 | 0.001 | 6.527 | 0.116 | 0.039 | 0.073 |
| 111 | Nitrogen metabolism | 39 | 0.34 | 2 | 0.044 | 3.118 | 1 | 0.285 | 0.061 |
| 212 | Cysteine and Methionine metabolism | 56 | 0.489 | 3 | 0.001 | 6.526 | 0.115 | 0.038 | 0.048 |
| 113 | Valine, Leucine and Isoleucine metabolism | 27 | 0.235 | 1 | 0.214 | 1.538 | 1 | 0.818 | 0.019 |
| 114 | Fructose and Mannose metabolism | 48 | 0.419 | 3 | 0.008 | 4.867 | 0.569 | 0.088 | 0.044 |
| 115 | Glyoxylate and dicarboxylate metabolism | 50 | 0.436 | 2 | 0.069 | 2.672 | 1 | 0.345 | 0.032 |
| 116 | D- Glutamine and D- Glutamate metabolism | 11 | 0.095 | 1 | 0.092 | 2.385 | 1 | 0.399 | 0.026 |
| 117 | Pentosoe phosphate pathway | 32 | 0.279 | 3 | 0.002 | 6.026 | 0.183 | 0.038 | 0.021 |
| 118 | Taurine and hyptaurine metabolism | 20 | 0.174 | 1 | 0.161 | 1.824 | 1 | 0.586 | 0.021 |
| 119 | Vitamin B6 metabolism | 32 | 0.279 | 1 | 0.245 | 1.402 | 1 | 0.748 | 0.019 |
| 220 | Starch and sucrose metabolism | 50 | 0.436 | 2 | 0.069 | 2.672 | 1 | 0.345 | 0.017 |
| 221 | Ascorbate and aldarate metabolism | 45 | 0.392 | 1 | 0.328 | 1.113 | 1 | 0.847 | 0.016 |
| 222 | Glutathione metabolism | 38 | 0.331 | 1 | 0.285 | 1.255 | 1 | 0.814 | 0.001 |
| 223 | Glycine, serine and threonine metabolism | 48 | 0.418 | 2 | 0.064 | 2.744 | 1 | 0.345 | 0.0004 |

**Figure S 13.** Summary of pathway analysis with MetaboAnalyst for prognosis of mortality. Each circle is a representative of a biological pathway and the size of the circle is enumerated based on the importance. **A:** summary of pathway analysis with MetaboAnalyst based on NMR data and **B:** GC-MS data. The number of the pathway corresponds to the number of the pathway shown in Tables S12 (NMR) and S13 (GC-MS).


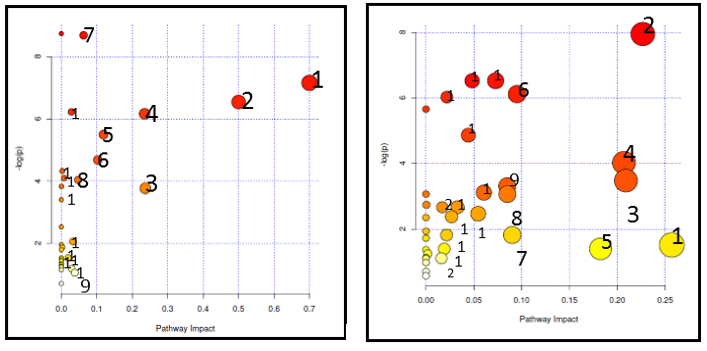


**Figure S13A** **Figure S13B**

**Table S13.** ROC curve analysis and linear regression for GC-MS, NMR, and APACHE II

|  | ROC Curve  Sensitivity (%) Specificity (%) AUC *p-*value | | | | Linear Regression  R2 *p-*value | |
| --- | --- | --- | --- | --- | --- | --- |
| GC-MS | 100 | 100 | 0.909 | <0.0001 | 0.909 | <0.0001 |
| NMR | 100 | 100 | 0.865 | <0.0001 | 0.860 | <0.0001 |
| APACHE II | 64 | 57 | 0.566 | 0.019 | 0.019 | 0.550 |

**Table S14**. A summary of PCA and OPLS-DA models regarding the number of metabolites/features for diagnosis and prognosis studies.

| Study | Number of Identified and Quantified Metabolites/Features | | | |
| --- | --- | --- | --- | --- |
|  | PCA | | OPLS-DA* | |
|  | NMR | GC-MS | NMR | GC-MS |
| H1N1 Pneumonia vs. Positive Bacterial Culture Pneumonia | 56 | 143 | 50 | 70 (known) |
| H1N1 Pneumonia vs. Ventilated ICU Controls | 55 | 135 | 55 | 68  (37 known) |
| H1N1 Survivors vs. H1N1 Nonsurvivors | 51 | 273 | 20 | 63   1. known) |

*The number of metabolites/features obtained based on the best OPLS-DA models that had highest Q2Y values

**Table S15:** Clinical and demographic characteristics of 21 H1N1 patients with laboratory-confirmed influenza H1N1 infection with Swine Influenza type a virus.

SD, standard deviation, ^a^ Data is No. (%) of subjects, unless otherwise indicated, LOS: Length of Stay, DBA: Days before admission, APACHE II: Acute Physiology and Chronic Health Evaluation II ICU scoring system, GI: Gastrointestinal, COPD: chronic obstructive pulmonary disease, CHD: coronary heart disease, PVD: peripheral vascular disease, DM: diabetes mellitus, IHD: Ischemic Heart Disease, CRI: Chronic renal insufficiency, SOB: shortness of breath, LOC: level of consciousness, ACS: Acute coronary syndrome. **^†^** The APACHE II score is for H1N1 and APACHE III is for positive bacterial culture, * reflects statistically significant difference in groups p < 0.05.

| Variables | Non Survived H1N1 Patients  (n= 7) | Survived H1N1 Patients  (n= 14) |
| --- | --- | --- |
| Age yrs. (mean ± SD) | 51.4 ± 18.3 | 50.2 ± 13.2 |
| Male/Female | 2/5 | 4/10 |
| BMI (mean ± SD) | 32.8 ± 11.7 | 35.2 ± 12.8 |
| Race  Caucasian  First nation | 4  2 | 11  3 |
| APACHE II | 23.2 ± 9.1 | 20.8 ± 8.3 |
| ICU LOS | 18.4 ± 7.8 | 18.5 ± 16.6 |
| Symptoms DBA | 5.2 ± 3.3 | 5.9 ± 3.2 |
| Comorbidity  Chronic heart failure  GI-diseases  COPD  Asthma  Neurological disorders  Other lung diseases  CHD  Arrhythmia  Hypertension  PVD  Cerebrovascular disease  Hematologic malignancies  Immunosuppression  DM  IHD_angina  Seizure  Scoliosis  CRI  Obesity  Cirrhosis | 2 (28) ^a^  2 (28)  1 (14)  2 (28)  2 (28)  1 (14)  0 (0)  0 (0)  4 (57)  1 (14)  0 (0)  2 (28)  1 (14)  3 (42)  0 (0)  1 (14)  0 (0)  2 (28)  1 (14)  1 (14) | 2 (14)  1 (7)  2 (14)  2 (14)  1 (7)  2 (14)  1 (7)  2 (14)  6 (42)  3 (21)  2 (14)  2 (14)  2 (14)  5 (35)  1 (7)  0 (0)  1 (7)  1 (7)  3 (21)  0 (0) |
| Smoker | 2 (28) | 5 (35) |
| Alcoholism | 2 (28) | 2 (14) |
| Pregnancy | 0 (0) | 2 (14) |
| Clinical manifestation  Fever > 38 ^★^  Headache  Myalgia’s  Weakness  Wheeze  Cough  Purulent Sputum  Pulmonary edema filtrate  SOB  Shock  [Altered LOC](http://www.google.ca/url?sa=t&rct=j&q=altered%20loc%20pathophysiology&source=web&cd=1&cad=rja&ved=0CC4QFjAA&url=http%3A%2F%2Fen.wikipedia.org%2Fwiki%2FAltered_level_of_consciousness&ei=Igr3UNr9KYSKiAKC5IHQDw&usg=AFQjCNGRfw8Tw5_GK6vD74Zkkem813h7Vw&bvm=bv.41018144,d.cGE)  ACS  CHD  Renal failure | 4 (57)  2 (28)  3 (42)  2 (28)  1 (14)  6 (85)  2 (28)  0 (0)  7 (100)  2 (28)  0 (0)  0 (0)  1 (14)  1 (14) | 13 (92)  2 (14)  7 (50)  6 (42)  3 (21)  13 (92)  6 (42)  2 (14)  13 (92)  3 (21)  1 (7)  0 (0)  1 (7)  1 (7) |
